# Deep learning reveals ecological decoupling and rate heterogeneity in Hymenoptera wing evolution

**DOI:** 10.1126/sciadv.aef0605

**Published:** 2026-10-14

**Authors:** Ancheng Peng, Shihan Guan, Laurent Albera, Régine Le Bouquin Jeannès, Huazhong Shu, Mathieu Boderau, André Nel, Bo Wang, Corentin Jouault, Frédéric Legendre

**Affiliations:** ^1^ Institut de Systématique, Évolution, Biodiversité (UMR 7205), Muséum national d’Histoire naturelle, CNRS, SU, EPHE-PSL, UA, Paris F-75005, France.; ^2^ State Key Laboratory of Palaeobiology and Stratigraphy, Nanjing Institute of Geology and Palaeontology, Chinese Academy of Sciences, Nanjing 210008, China.; ^3^ Laboratory of Image Science and Technology (LIST), School of Computer Science and Engineering, Southeast University, Nanjing 210096, China.; ^4^ Centre de Recherche en Information Biomédicale Sino-français (CRIBs), Inserm, Rennes F-35000, France.; ^5^ Univ Rennes, Laboratoire Traitement du Signal et de l’Image (LTSI), UMR 1099, Rennes F-35000, France.; ^6^ Oxford University Museum of Natural History, University of Oxford, Oxford OX1 3PW, UK.

## Abstract

Quantifying biomorphological evolution and integrating phenotypic data into large-scale ecological and evolutionary studies are often limited by the scalability, subjectivity, and poor interpretability of existing methods. We introduce HAIKA (Hierarchical Attention-based Interpretable K-dimensional Autoencoder), a self-supervised residual channel-attention autoencoder that extracts continuous morphological representations from unlabeled standardized images and highlights the image regions that contribute to those representations. Applied to insect forewings across broad pterygote lineages, HAIKA outperformed most baseline deep learning models and conventional descriptors in clustering, dimensionality reduction, and morphological embedding. When applied to Hymenoptera (i.e., ants, bees, and wasps), the HAIKA-derived continuous characters improved morphology-based phylogenetic reconstruction and illuminated the evolutionary dynamics of wing shape and venation. Our results reveal that wing morphological evolution in Hymenoptera proceeds at decoupled rates across shape and venation modules and is not consistently aligned with key ecological transitions such as parasitoidism or eusociality. HAIKA provides a scalable, image-based route for phenotypic analyses, broadly applicable across diverse taxa and to both 2D and 3D datasets.

## INTRODUCTION

Despite the explosive growth of molecular datasets over the past few decades, morphological data remain fundamental to evolutionary biology and systematics (*1*–*4*). Not only is morphology essential for integrating and making sense of fossils, but it is also key to understanding major evolutionary innovations, such as the emergence of wings in insects (*5*). This enduring importance has spurred methodological advances in data acquisition and analysis [e.g., (*6*–*8*)]. However, the process is still burdened by several bottlenecks. Data collection remains slow and labor-intensive, relying heavily on manual landmarking and outline tracing, even when supported by semiautomated tools [e.g., (*9*, *10*)]. Furthermore, many existing analytical pipelines simplify complex morphological diversity, resulting in reduced representations and an avoidable loss of biologically meaningful information (*11*, *12*).

To overcome these challenges, the scientific community has increasingly embraced artificial intelligence, especially deep learning approaches (*13*–*17*). This shift from manually designed image processing to data-driven methods has enabled high-throughput phenotyping across micro- and macroevolutionary scales (*14*, *16*). Nonetheless, several obstacles still hinder the widespread adoption of deep learning approaches in comparative biology. These limitations include high computational demands (*18*), restricted generalizability across broad taxonomic ranges (*19*), and, critically, a lack of biological interpretability, where features driving classification are opaque to the user (*20*).

We introduce HAIKA (Hierarchical Attention-based Interpretable K-dimensional Autoencoder), a self-supervised deep learning workflow (Fig. 1) developed to advance morphological analysis. HAIKA integrates well-established encoder-decoder, residual, and squeeze-excitation channel-attention components into a comparative morphology pipeline. HAIKA adapts these existing components to extract continuous morphological representations from unlabeled images and applies them to systematics, phylogenetic inference, and macroevolutionary analysis. This workflow reduces the need for manual annotation and prior landmark definitions for each new dataset. We demonstrate this potential through a series of case studies focusing on insect wings.

**Fig. 1.**
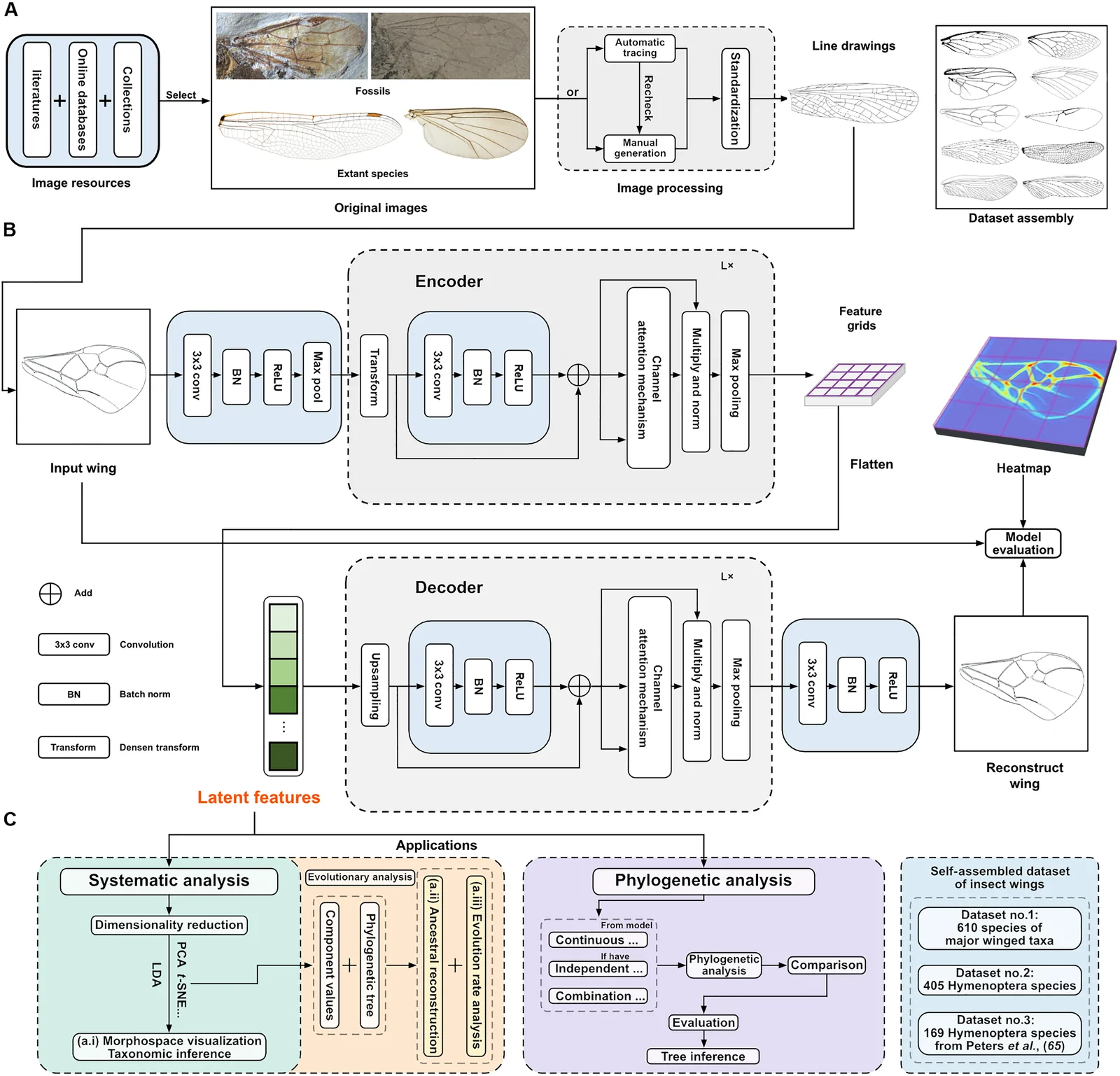
Workflow of HAIKA-based morphological representation learning and downstream comparative analyses. (**A**) Construction of the standardized insect wing image dataset, including data collection, curation, preprocessing, and subdataset assembly. (**B**) Architecture of HAIKA, which combines an encoder-decoder backbone with residual connections and channel-attention modules to extract continuous morphological representations from standardized images. (**C**) Application of HAIKA-derived latent features to three downstream datasets for morphospace analysis: taxonomic attribution, phylogenetic reconstruction, and macroevolutionary interpretation.

Insect wings provide an ideal testing ground for this framework. As a key innovation that propelled insect diversification over the past 350 million years, insect wings have accumulated remarkable morphological complexity (*5*, *21*–*24*). Wing shape and venation patterns, in particular, are essential diagnostic features that provide crucial information for the systematics and evolutionary studies of extant and fossil species across broad taxonomic and phylogenetic scales [e.g., (*25*–*28*)].

We trained HAIKA on a dataset of over 1000 insect forewings, spanning most major winged-insect lineages. Our extensive validation procedure demonstrated that HAIKA mostly outperforms 14 fine-tuned baseline models across established standard clustering and dimensionality reduction metrics (Fig. 2). We then applied HAIKA to three distinct wing datasets to evaluate its utility in systematics and evolutionary biology. In Case 1, HAIKA extracts complex, cross-taxon morphological features, highlighting its potential for automated taxonomic attribution. In Case 2, we showed that HAIKA-derived representations contain phylogenetically relevant signal and can improve morphology-based tree inference in the present dataset. In Case 3, we leveraged HAIKA-extracted features to investigate the evolutionary history of Hymenoptera wings, testing for heterogeneous evolutionary rates in shape and venation modules and relating morphological transitions to major ecological strategies [e.g., (*28*–*33*)]. Although our study focuses on 2D insect imagery, HAIKA’s architecture is extensible to other morphological structures, taxa, and imaging modalities, including 3D data.

**Fig. 2.**
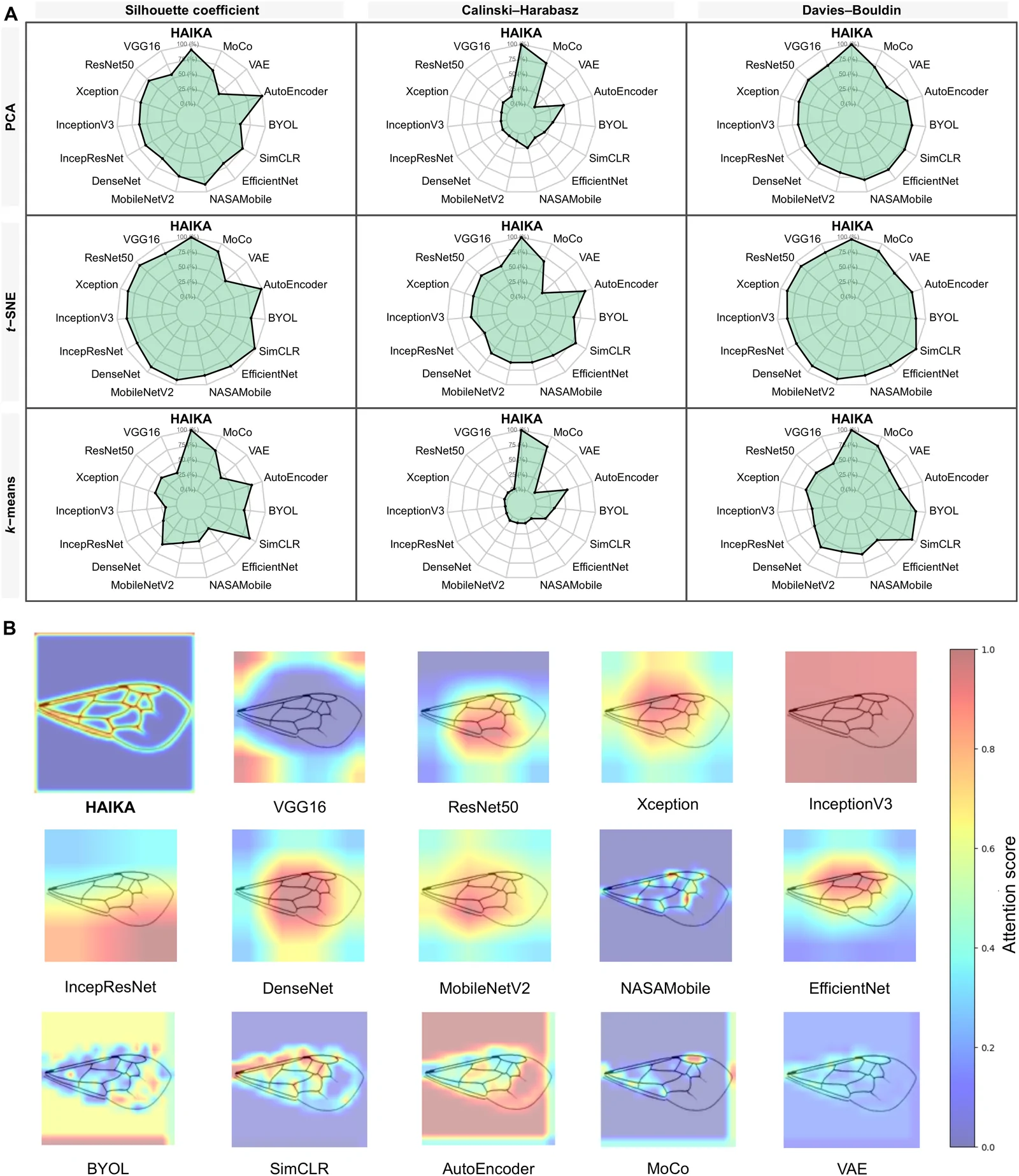
Benchmarking and visual plausibility assessment of HAIKA-derived representations. (**A**) Performance of HAIKA and 14 baseline deep learning networks across clustering and dimensionality-reduction metrics. Scores were rescaled relative to the best value for each metric, with 100% indicating the best-performing method; higher values are better for the SC and CH indices, whereas lower values are better for the DB index. Raw metric values are provided in table S2. (**B**) Attention and saliency visualizations across models. HAIKA attention is concentrated on morphologically informative wing regions, including the contour, veins, and vein junctions, whereas several baseline models emphasize membrane regions.

## RESULTS

The workflow of our study is illustrated in Fig. 1, including the dataset preprocessing (Fig. 1A), model architecture (Fig. 1B and table S1), and downstream evolutionary biology and systematics applications (Fig. 1C). For information on the design ideals, motivations, and technical details of the model, refer to Materials and Methods.

### Model evaluation

Before conducting systematics and evolutionary biology case studies, we evaluated the performance of HAIKA against 14 fine-tuned networks (nine supervised and five self-supervised, representing the most widely used deep learning models currently) across three established clustering and dimensionality reduction metrics (Fig. 2A and table S2). We also evaluated four conventional morphological descriptors as complementary, non–deep-learning benchmarks (table S2).

Across all unsupervised tests, including *k*-means clustering, principal components analysis (PCA), and *t*-distributed stochastic neighbor embedding (t-SNE), HAIKA produced clusters with tighter within-cluster cohesion and greater between-cluster separation than most baseline models, yielding more structured clusters and clearer separation within the morphospace. In the *k*-means test, HAIKA achieved optimal values for all three metrics: a silhouette coefficient (SC) of 0.258 (baseline best, SimCLR: 0.227), a Calinski-Harabasz (CH) score of 798.1 (baseline best, MoCo: 649.9), and the lowest Davies-Bouldin (DB) index of 1.074 (baseline best, SimCLR: 1.167). In the PCA metrics, HAIKA yielded the highest CH score (817.0 versus baseline best, Autoencoder: 404.3), the lowest DB index (1.000 versus baseline best, NASAMobile: 1.258), and the second-highest SC score (0.239 versus baseline best, Autoencoder: 0.262). For the *t*-SNE embedding, HAIKA achieved the highest SC (0.418 versus baseline best, Autoencoder: 0.412), the highest CH score (798.1 versus baseline best, Autoencoder: 697.1), and the second-lowest DB score (0.821 versus baseline best, Autoencoder: 0.793). We also observed that self-supervised baseline methods typically produce better scores than supervised models. Repeating HAIKA training across five random seeds yielded similar metric values (tables S9 and S10), reducing the likelihood that the benchmark reflects a single favorable initialization.

These quantitative results indicate that the model-derived features retain substantial information from the original images (Fig. 2B). Attention heatmaps for HAIKA, NASAMobile, and Grad-CAM++ saliency maps generated for the remaining 13 baseline models showed that HAIKA consistently focused on wing-informative structures, including the wing margins, venation, and vein junctions. Conversely, most baseline models focused on uninformative membrane regions. Furthermore, a region-level concordance analysis showed that the dominant HAIKA attention matched the expert-recognized midwing region in 203 out of 210 Hymenoptera images (96.7%; Supplementary Text and fig. S8). These results support the morphological plausibility of the learned representation.

### Taxonomic attribution: HAIKA captures taxonomically relevant morphological patterns from insect wings (Case 1)

As suggested by the attention visualizations, HAIKA extracted taxonomically informative wing features in an unsupervised manner at two taxonomic scales: (i) a broad dataset of 610 wing images spanning all major winged insect groups, including extant and fossil taxa, and (ii) a focused dataset of 405 hymenopteran species representing most extant subfamilies and major genera. In the broad insect dataset, the first two principal components (PCs) of HAIKA’s latent features accounted for 86.2% of the variance (PC1: 79.5%, PC2: 6.7%). The four focal clades (Palaeoptera, Acercaria, Polyneoptera, and Holometabola) formed distinct clusters with separate centroids (Fig. 3A), despite some expected overlap due to convergent venation patterns (e.g., Mecoptera resembling some Polyneoptera). Permutational multivariate analysis of variance (PERMANOVA) indicated significant morphological differentiation among all four clades (*P* < 0.001), and PERMDISP tests detected significant heterogeneity in within-group dispersion (*F* = 31.45, *P* = 0.0001; table S11), consistent with the broad morphological disparity spanning these lineages. Linear discriminant analysis (LDA) achieved 85.6% accuracy (84.1% under jackknifing). Removing residual links and channel-attention mechanisms reduced accuracy to 77.4% (75.6% under jackknifing), supporting the contribution of these components to feature extraction.

**Fig. 3.**
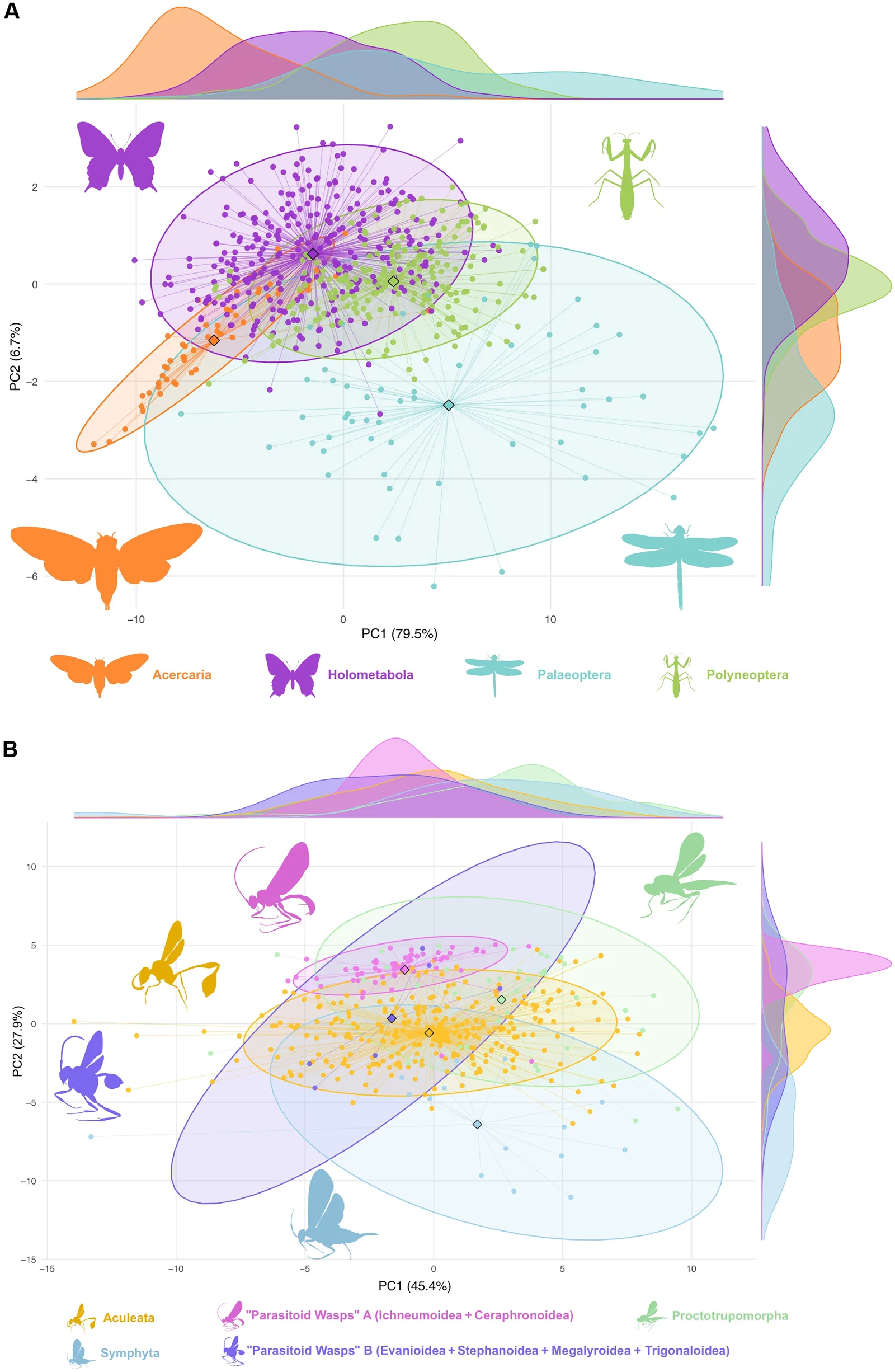
HAIKA-derived morphospaces recover structured wing variation across insect and hymenopteran groups. (**A**) Morphospace of major winged-insect clades in dataset no. 1, grouped according to Nel *et al.* (*119*), Misof *et al.* (*120*), and Wipfler *et al.* (*121*). (**B**) Morphospace of major Hymenoptera groups in dataset no. 2, grouped according to Ronquist *et al.* (*34*), Peters *et al.* (*65*), Blaimer *et al.* (*58*), and Zhang *et al.* (*59*). Points represent species-level wing embeddings projected onto the first two PCs. Marginal density curves show the distribution of each group along the corresponding morphospace axis. Ellipses represent 95% confidence regions, and diamonds indicate group centroids. Raw matrices are available in Zenodo (10.5281/zenodo.22715169). Insect silhouettes were obtained from PhyloPic (http://phylopic.org), released under the Creative Commons Public Domain Dedication (CC0 1.0; https://creativecommons.org/publicdomain/zero/1.0/), or drawn by M.B. [Acercaria; Hymenoptera after illustrations in Goulet and Huber (*127*)].

The hymenopteran dataset exhibited analogous patterns of taxonomic clustering (Fig. 3B). The first two PCs explained 73.3% of the total variation (PC1: 45.4%, PC2: 27.9%), and the resulting morphospace reflected established taxonomic groupings, with “symphytan” superfamilies and apocritan infraorders forming distinct clusters (*P* < 0.001). PERMANOVA detected multivariate differences between these groups (*P* = 0.0001), whereas the distance between the morphologically similar “parasitoid” wasps B (Evanioidea + Stephanoidea + Megalyroidea + Trigonaloidea) and Aculeata was, as anticipated, not significant (*P* = 0.2801). Because PERMDISP also detected significant dispersion heterogeneity (table S11), these PERMANOVA results are treated as evidence of structured wing variation rather than as centroid separation alone. LDA accuracy reached 83.9% (78.7% under jackknifing), further supporting the presence of taxonomically informative morphological signals. Feature extraction was performed in a fully unsupervised manner, and taxonomic labels were introduced only at the classification stage.

### Deriving morphological data for phylogenetic analyses with HAIKA (Case 2)

To assess the phylogenetic utility of HAIKA-derived data, we performed both constrained and unconstrained parsimony analyses of Hymenoptera using three morphological datasets: (i) the discrete character matrix of Ronquist *et al.* (*34*), (ii) a continuous matrix of 64-dimensional image-based representations derived from HAIKA, and (iii) a combined matrix incorporating both sources. In analyzing the latter, we applied a weighting scheme to limit the influence of the continuous character matrix derived from HAIKA and explored the impact of character dependence by including and excluding wing-related characters from the discrete matrix (see details in Materials and Methods). Summary metrics are presented in Table 1 and topologies in figs. S1 to S3.

**Table 1.** Summary of different indicators for the three morphological matrices: discrete, continuous, and combined. Indicators are reported for unconstrained/constrained analyses.

Matrices	Discrete	Continuous	Combined
No. of equiparsimonious trees	>10,000/>10,000	5/1	1302/762
Tree length	1245/1280	92/96	1345/1349
Consistency index	0.355/0.345	0.921/0.883	0.391/0.390
Retention index	0.723/0.710	0.880/0.813	0.723/0.722
Nodes recovery (out of 23 / out of 17)	11 (47.8%)/11 (64.7%)	11 (47.8%)/10 (58.8%)	19 (82.6%)/14 (82.3%)

The continuous HAIKA-derived matrix showed substantially higher consistency and retention indices (∼0.9 versus ∼0.3 to 0.4) and produced more fully resolved trees, whereas analysis of the discrete matrix alone resulted in a notably unresolved topology. While both separate matrices recovered a similar proportion of phylogenetic nodes (∼50 to 60%), the combined matrix clearly performed best, recovering ∼80% of the nodes (Fig. 4: nodes 1 to 3, 5 to 6, 9, and 20). These improvements are biologically meaningful: Notably, the analysis of the combined dataset was the only one that successfully recovered the monophyly of Apocrita (node 1), a central and well-established clade in hymenopteran systematics. Bootstrap support also increased at several deep nodes when continuous HAIKA-derived characters were included, whether alone or in combination (Fig. 4: nodes 4, 7, 14, and 16), supporting the finding that HAIKA-derived characters can enhance topological robustness in this dataset.

**Fig. 4.**
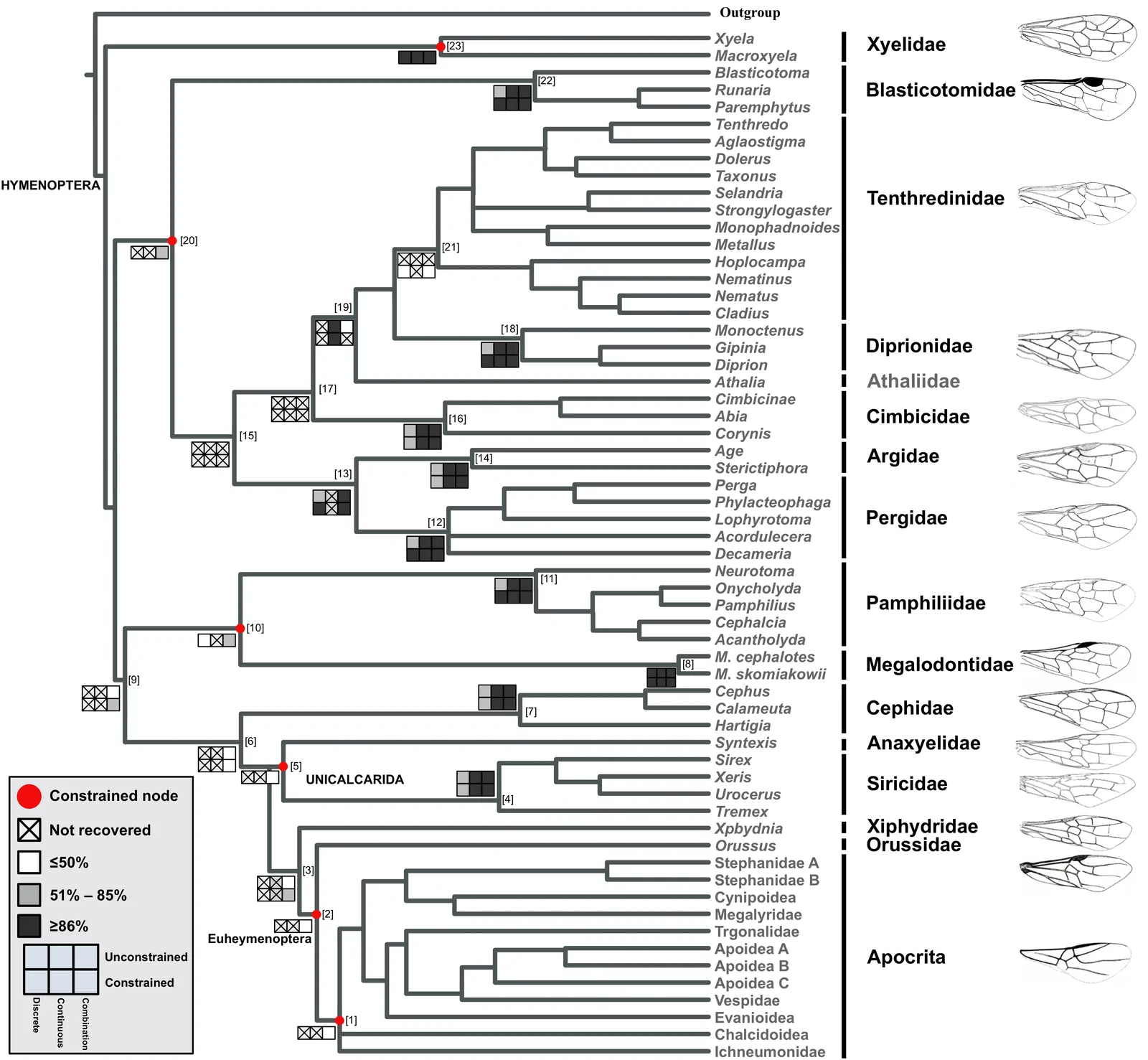
Phylogenetic performance of discrete, HAIKA-derived continuous, and combined morphological matrices in Hymenoptera. Summary of parsimony analyses for the discrete character matrix, the HAIKA-derived continuous matrix, and the combined matrix, plotted on the reference phylogeny of Ronquist *et al.* (*34*). Node numbers correspond to the deep nodes evaluated in Results. Red dots indicate nodes constrained in the constrained analyses following Ronquist *et al.* (*34*). Square grids summarize whether each matrix recovered the corresponding node under unconstrained and constrained analyses. Full strict consensus trees for the three matrices are provided in figs. S1 to S3.

### Wing evolution dynamics in Hymenoptera (Case 3)

We extracted a HAIKA-based morphological matrix for Hymenoptera and characterized the main axes of wing variation using PCA. The first two PCs accounted for 74.5% of the total variance (PC1: 42.5%, PC2: 32.0%). Multivariate multiple linear regressions revealed that PC1 primarily reflects wing shape [associated with aspect ratio (AR) and standard mean chord (SMC); table S7], whereas PC2 captures venation complexity (strongly linked to number of cells, distal extension of veins, and pterostigma state; table S8). Distal extension of veins was the only trait related to both PCs.

Both PC1 and PC2 exhibited a strong phylogenetic signal (Pagel’s λ ≈ 1, *P* < 0.001; Blomberg’s *K* > 1.5, *P* = 0.001; table S3). A Brownian motion model best explained their evolution, but the estimated rates differed markedly: PC1 evolved ∼1.7× faster than PC2 (σ^2^ = 58.84 versus 34.65, *P* = 0.001; table S3 and fig. S7).

Ancestral reconstructions showed that changes exceeding the mean difference of ancestral state reconstruction (ASR) value between two adjacent nodes (Δ_mean_ = 0.37 and 0.43 for PC1 and PC2, respectively; tables S5 and S6) were predominantly concentrated in symphytan groups, parasitoid lineages, and the predatory Sphecidae (Fig. 5). Many of the 29 additional shifts coincided with major ecological transitions (parasitoidism, eusociality, and pollen collecting) or functional innovations (wasp-waist). We treat the Δ_mean_ threshold as a transparent heuristic metric for identifying above-average morphological changes, not as a formal shift-detection model or a test of ecological causality.

**Fig. 5.**
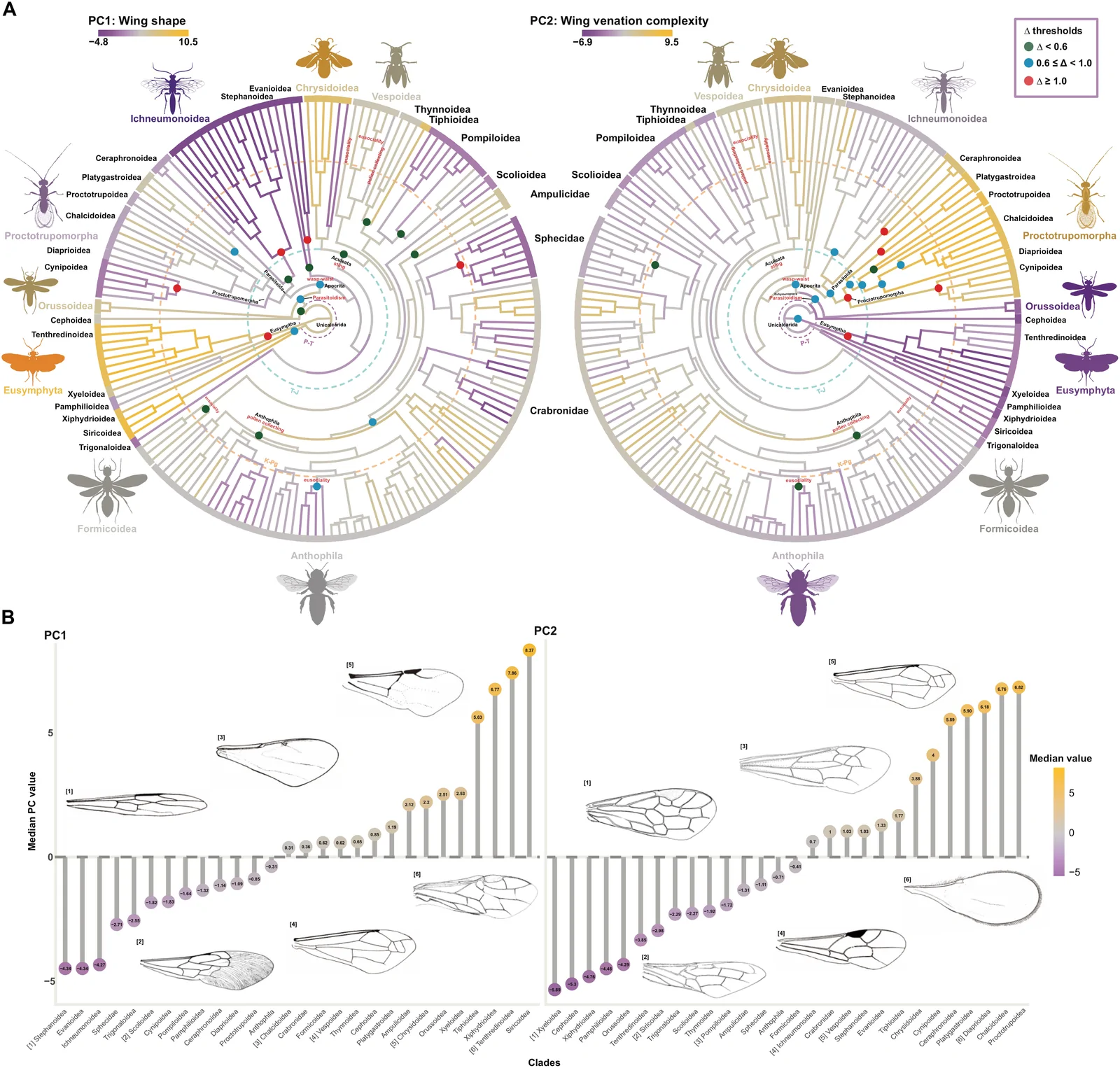
Ancestral reconstructions of HAIKA-derived wing shape and venation axes in Hymenoptera. (**A**) Maximum-likelihood ancestral state reconstructions of wing shape (PC1) and venation complexity (PC2) mapped onto the time-calibrated Hymenoptera phylogeny using continuous character mapping. Color gradients represent reconstructed trait values along branches, and marked nodes indicate changes exceeding the mean node-to-node difference (Δ_mean_) for each PC axis. Major ecological and functional transitions are annotated for comparison. (**B**) Distribution of PC1 and PC2 scores across major hymenopteran clades. Black numbers indicate clade medians, and numbered wing illustrations correspond to the clades listed below the plots. Raw matrices, ancestral reconstruction values, and statistical summaries of the five wing-related characters are available in Zenodo (10.5281/zenodo.22715169) and tables S3 to S7. Insect silhouettes were obtained from PhyloPic (http://phylopic.org), released under the Creative Commons Public Domain Dedication (CC0 1.0; https://creativecommons.org/publicdomain/zero/1.0/), or drawn by A.P. [Hymenoptera after illustrations in Goulet and Huber (*127*)].

Symphytan clades exhibited above-average shifts in both wing shape and venation, characteristic of their broad, densely veined wings. However, the magnitude of these shifts may partly reflect subsequent reductions within Apocrita. Key transitions at the origins of Euhymenoptera (known as “Vespina”) and Apocrita were associated with decreases in venation complexity and a narrowing of the wing base. Multiple independent simplification events in venation were detected during the radiation of parasitoid wasps, especially in Ichneumonoidea, Ceraphronoidea, and Proctotrupomorpha, and were often accompanied by shape changes.

In contrast, early-diverging Aculeata exhibited limited shifts in wing morphology, with later changes concentrated in Vespoidea, Tiphioidea, Ampulicidae, Sphecidae, Formicoidea, and bee-related nodes. Venation remained relatively stable, with localized modifications in Vespoidea, Sphecidae, and Anthophila. The origin of bees was associated with moderate changes in shape and minimal changes in venation. Nodes linked to eusociality and pollen collection showed variable but mostly moderate shifts.

## DISCUSSION

### HAIKA outperforms other deep learning networks in systematics

Progress in understanding morphological evolution is directly limited by our ability to acquire and quantitatively analyze large morphological datasets (*35*–*37*). HAIKA was designed to facilitate high-throughput extraction of morphological information directly from unlabeled image data, reducing the need for a priori landmark identification and for explicit character-state definition once standardized images have been assembled.

HAIKA’s design, based on a self-supervised encoder-decoder architecture, sidesteps two limitations of supervised or hybrid models: reliance on preassigned labels and dependence on fixed taxonomic hierarchies. First, supervised models can suffer from “label noise” or “label error” (*38*, *39*), leading to degraded performance and poor generalization, especially when labeled data are scarce (*18*). Second, models constrained by predefined taxonomic hierarchies are also limited by the accuracy and resolution of those hierarchies [e.g., (*20*, *40*)], which can be problematic for fossils, undescribed taxa, or groups with unstable systematic frameworks [e.g., (*41*)]. By contrast, HAIKA learns from standardized images, as the reconstruction and feature-extraction step proceeds without requiring the investigator to specify landmarks, define character boundaries, or assign character states, subsequently reducing dependence on prior character definitions. Consistent with this design, the stronger performance of self-supervised baselines relative to supervised classifiers should be interpreted in relation to the evaluation target. Because the downstream metrics assess unsupervised structure in the feature space, self-supervised objectives are more directly aligned with the evaluation framework than purely supervised classification objectives. This alignment does not by itself establish biological superiority, but it supports the use of self-supervised representation learning for label-free morphological exploration.

We demonstrate that HAIKA effectively learns morphologically informative latent features and achieves high classification accuracy directly from the standardized morphological data. We show that HAIKA’s focus aligns with expert-defined landmarks, supporting the view that its latent features capture relevant morphological characters. While supervised models can also achieve high accuracy, this performance is not always coupled with the extraction of biologically meaningful features [e.g., (*14*, *20*)]. We address this by integrating attention mechanisms with residual connections, which enables HAIKA to extract continuous, biologically consistent traits while maintaining training stability. This design provides a high degree of attentional transparency, aligning with the goals of interpretable AI (*42*). It allows HAIKA not only to generate structured representations but also to visualize the anatomical regions associated with those representations (Fig. 2B). This interpretability supports an iterative workflow where experts can rapidly adjust training parameters based on immediate feedback from the model’s attention maps. This dynamic feedback loop represents a shift from the slower refinement processes of traditional and opaque black-box approaches.

While our primary case studies focus on 2D images of insect wings, this workflow can be extended to other morphological structures, taxa, and data modalities. Its potential application to 3D volumetric data from sources like μCT scanning is particularly promising where traditional landmarking is challenging (*43*, *37*). Our preliminary analyses, which incorporate architectural modifications for volumetric data, confirm that HAIKA successfully achieves cohesive clustering on both the primate and standard 3D datasets (figs. S5 and S6). However, additional validation on anatomically complex 3D datasets is still needed before applying the same workflow.

### Contribution of HAIKA to phylogenetic tree reconstruction

While molecular data are essential for phylogenetics, driving outstanding progress in deciphering the tree of life, they face inherent limitations (e.g., conflicts between loci and unavailability for fossils) that necessitate combination with other data sources, notably morphology (*1*, *2*, *44*, *45*). Although inferring organismal relationships from morphology is a long-standing objective in systematics, recent deep learning applications in this domain have yielded mixed results: Some studies successfully capture phylogenetic signals at broad taxonomic levels (*20*, *46*), yet others report limited phylogenetic information despite achieving high classification accuracy (*40*, *47*).

Our investigation into Hymenoptera phylogenetic relationships suggests that HAIKA-derived wing representations contain phylogenetically relevant signals. The combined phylogenetic analysis revealed that the continuous matrix improved resolution and support—relative to the discrete matrix alone—notably recovering the monophyly of Apocrita and strengthening the support for several deep nodes. These results indicate that HAIKA can provide an insightful and scalable source of morphological information for phylogenetic inference. However, because the latent dimensions are jointly learned and may be correlated, their use requires explicit weighting decisions, sensitivity analyses, and careful separation of unconstrained and partially constrained results.

Furthermore, as illustrated in Case 3, the representations generated by HAIKA can be subjected to dimensionality reduction and interpreted in conjunction with specific morphological features. This allows researchers to formulate quantitative hypotheses about the rates and patterns of morphological evolution. The data-driven trait axes may be useful in future evolutionary models, such as Bayesian neural networks that link traits to speciation and extinction dynamics (*48*). Specifically, the data-driven “trait modules” inferred by HAIKA provide a complementary approach for formulating and testing macroevolutionary hypotheses, for instance, about the factors driving the main axes of evolution in morphospace.

### Complex and asynchronous evolution of wing traits in Hymenoptera

The downstream application of HAIKA provides an exploratory insight into the macroevolutionary dynamics of Hymenoptera wings. The evolution of insect wings has long been recognized as a product of complex interactions among ecological transitions, functional demands, and developmental and phylogenetic constraints (*49*–*53*). Classical hypotheses in Hymenoptera suggest that parasitism drives wing miniaturization and venation simplification (*29*, *54*, *55*), while predation and foraging specialization shape overall flight performance (*28*, *56*, *57*). Our study used HAIKA-derived continuous characters to explore whether nodes with major morphological changes in wing forms (those with high Δ) are consistently coupled to these key ecological traits or functional transitions.

Two findings are that major morphological changes are not consistently associated with major changes in life history traits, and the evolutionary patterns and rates of wing shape (PC1) and venation complexity (PC2) often exhibit asynchronous dynamics. Starting at the tree root, the highest changes (Δ) are observed in symphytan lineages without clear ecological or functional coupling. Considering that the early relationships within Symphyta remain unsettled (e.g., Eusymphya versus a basal Xyelidae), node-specific Δ within these lineages are expected to vary modestly across different topologies (*58*, *59*). These initial changes may represent either the early establishment of the hymenopteran wing bauplan, characterized by relatively broad bases, low ARs, and dense venation, or initial morphological exploration during early radiation (*45*, *58*, *60*, *61*). Because no outgroups were sampled, these elevated Δ values may alternatively reflect departures toward the tapered, venation-reduced wings typical of Apocrita (Fig. 5).

At the Euhymenoptera node (Parasitoidism: Orussoidea + Apocrita), elevated Δ values coincided with the emergence of a parasitoid lifestyle. However, the magnitude of Δ at this node is smaller than in subsequent apocritan radiations, suggesting that early parasitoidism on sedentary hosts imposed effective, albeit limited, selection on flight performance (*62*). Orussids, for instance, rely on vibrational cues to locate wood-boring hosts, which may reduce the selective pressure for aerial searching innovation (*63*, *64*). This early transition may have established the ecological and metabolic conditions for subsequent diversification (*64*), while more pronounced wing morphological changes appeared during later apocritan radiations (*45*, *58*).

We observed a major, coupled change in wing morphology coincident with the key functional innovation at the origin of Apocrita (the wasp-waist). The evolution of the wasp-waist enhanced metasomal mobility and flight agility, imposing distinct aerodynamic constraints that favored narrower, higher–aspect-ratio wings with simplified venation (*53*–*56*, *58*, *65*). This wing pattern recurs across major parasitoid radiations, with multiple lineages (e.g., Ichneumonoidea, Ceraphronoidea, Evanioidea, and Cynipoidea) showing above-mean Δ values, likely indicating repeated, convergent responses to similar ecological pressures (*45*, *58*, *66*). These convergent trends may reflect distinct adaptive trade-offs, such as endurance versus maneuverability and structural support versus flexibility (*28*, *56*). For example, ichneumonoids evolved high–aspect-ratio wings suited for long-distance host searching (*67*), while miniaturized Ceraphronoidea emphasized venation reduction for flexibility in low–Reynolds-number flight (*28*, *29*). These coordinated morphological trends also coincide with recently revealed genomic signatures of parasitoid evolution (*68*), which possibly imply a functional integration between wing design, physiology, and parasitic ecology.

In contrast, Aculeata are characterized by the presence of a functional sting, and their ingroup lineages show overall limited changes, suggesting a relative decoupling between these major eco-behavioral innovations and large-scale alterations in wing morphology (Fig. 5). One possible explanation is ecological or phylogenetic inertia, in which the ancestral parasitoid wing design was already optimized for agile and generalized flight. Stinging and foraging behaviors may have evolved without imposing fundamentally distinct aerodynamic demands (*28*, *56*, *69*). Consequently, the morphospace accessible to Aculeata was likely preoccupied by Parasitoida, favoring fine-scale adjustments rather than the exploration of alternative configurations [e.g., (*48*, *70*–*72*)]. This situation aligns with the genomic stability observed in Aculeata (*68*). It parallels findings in other specialized fliers such as hoverflies, where stabilizing selection maintains morphological stasis despite ecological diversification (*53*). Nonetheless, ongoing fine-scale morphological tuning associated with ecological partitioning and local adaptation is still visible in certain subclades like Sphecidae and some apoid lineages (Fig. 5). However, this inference is based on winged individuals and requires caution in groups with apterous morphs or caste polymorphism.

Similarly, eusocial nodes (Anthophila/bees, Formicidae/ants, and Vespidae/wasps) exhibit modest wing morphological changes within the Aculeata, possibly because the adaptive basis of eusociality lies mainly in reproductive division of labor, communication, and nest architecture, rather than individual flight performance (*56*, *69*, *73*–*75*). However, the evolution of pollen collection presents divergent patterns across Aculeata. In bees, the biomechanical demands of carrying heavy pollen loads and long-distance foraging likely impose selective pressures on wing architecture (*76*). In contrast, many predatory Vespidae lineages show minimal change, likely because their flight ecology remains within the functional range of ancestral predatory flight (*77*–*79*). This differential response highlights that morphology-ecology coupling is contingent not only on the ecological transition itself but also on the degree of specialization, the phylogenetic starting point (ancestral wing design), and the biomechanical demands imposed by new behaviors.

Additionally, our results suggest that the hymenopteran wing shape (PC1) and venation complexity (PC2) evolved asynchronously and at different rates. Venation evolves more slowly, which may reflect structural constraints, as excessive simplification could compromise structural integrity and aerodynamic efficiency, consistent with prior morphological constraints identified in hymenopteran forewings (*28*). Overall, our findings support the complexity and contingency of Hymenoptera wing evolution, demonstrating that dramatic morphological shifts do not always accompany major ecological and behavioral innovations, and that the interplay of function, lifestyle, and phylogenetic history may constrain the trajectory of wing diversification.

### Summary, future work, and limitations

The development of HAIKA represents a step toward scalable feature extraction in phenomics. By combining a self-supervised deep learning architecture with visual plausibility checks and downstream comparative analyses, HAIKA reduces dependence on manually defined landmarks and character states. Its application to Hymenoptera indicates that image-derived descriptors can contain taxonomic and phylogenetically relevant information and can describe asynchronous evolution of wing shape and venation. These results support the use of HAIKA as a reproducible exploratory framework for integrating image-derived morphological characters into systematic and evolutionary studies.

While demonstrating advantages, we also recognize that our work has limitations and space for future expansion. Regarding limitations, first, preprocessing remains an essential component of the workflow because, for example, poor image quality, low contrast, extensive artifacts, or substantial occlusion can reduce the generalization performance of the model. This concern is not specific to HAIKA, as data cleaning is a fundamental requirement for any model and must be addressed before training. This limitation should be disclosed for any application involving heterogeneous datasets (such as those from different museums, illustrators, and equipment), and we strongly recommend implementing source-effect diagnostics when provenance metadata are available. That said, mature computer vision algorithms for automated alignment, geometric correction, and keypoint-based normalization are now widely available. These approaches may eventually replace the manual steps currently required and provide a more scalable preprocessing pipeline. Second, HAIKA’s optimization objective is a reconstruction loss [using mean squared error (MSE) for continuous pixel values] rather than an explicit classification objective. While fully supervised end-to-end classifiers (e.g., fine-tuned models using ResNet or EfficientNet with cross-entropy loss) achieve higher accuracy on pure classification tasks, the latent representations generated by HAIKA are effective for downstream classification via LDA. Although the reconstruction of latent representations has practical value for exploratory and downstream analyses, the reconstruction itself cannot prove its biological significance and still needs to be cross-validated with a variety of statistical methods. Furthermore, the autoencoder architecture is inherently scalable to supervised and semisupervised paradigms (*80*, *81*). Such a hybrid approach would maintain the interpretability afforded by reconstruction-based models, such as attention maps and smooth transitions along morphological gradients, while simultaneously improving classification performance when needed.

Several avenues can extend the HAIKA framework and broaden its applicability. One area concerns the handling of geometric and rotational variations, as the encoder may lose generalization performance when processing data with inconsistent orientations or severe distortions. We envision that incorporating rotation-equivariant architectures, such as O(2)-equivariant steerable convolutional networks (*82*, *83*), would allow the encoder to learn stable feature representations without explicit alignment. A second direction is to explicitly explore treating the decoder of our framework as a generative model for wing morphology. Rather than merely reconstructing the compressed embedding, future variants could equip the decoder with stronger generative capacity, for example, through diffusion-based decoders, StyleGAN-like generators, or latent diffusion models (*84*–*86*), to synthesize clear and high-fidelity reconstructions of the original wing shapes and venation patterns. By requiring the model to regenerate biologically realistic wings with fine-scale structural details, the learned latent representations are expected to become more tightly constrained by anatomical regularities and, consequently, more biologically meaningful for downstream analyses of morphological variation and evolutionary dynamics. A third direction is to further integrate other large AI models to build automated phenotypic preprocessing pipelines. Foundation models, such as the Segment Anything Model [SAM; (*87*)], provide generalizable segmentation across imaging domains. In our wing-shape pipeline (also see the quantification method of wing shape parameters part in the Supplementary Materials), SAM-based contour segmentation achieved a mean IoU of 0.9 against expert annotations and an average processing time of 1.2 s per image on an NVIDIA RTX A5000 GPU. Lastly, incorporating continuous image-derived representations into Bayesian total-evidence frameworks remains a priority, because our present phylogenetic test used maximum parsimony as a pragmatic choice under current modeling constraints [see also the relevant discussion in (*14*)].

## MATERIALS AND METHODS

### Motivation and overall architecture

To address the persistent challenge of high-throughput phenotyping in eco-evolutionary studies, we developed HAIKA (Fig. 1B and fig. S4), a deep learning workflow designed to extract features from complex, unlabeled morphological datasets. A key design principle was the capacity to accommodate datasets of varying types (e.g., 2D images and 3D data stacks), quality, and size. To this end, HAIKA is built upon a self-supervised encoder-decoder architecture, which has proven useful in computer vision, medicine, and microscopy (*83*, *88*–*90*). In HAIKA’s workflow, the encoder transforms high-dimensional image data into a low-dimensional latent feature space: a set of quantitative and continuous morphological vectors. The dimensionality of this vector group is adjustable depending on the complexity of the dataset. The decoder attempts to reconstruct the original image from this vector, so reconstruction fidelity indicates that image information is retained under compression.

To improve feature propagation and region weighting, we integrated two architectural components. First, a squeeze-excitation channel-attention mechanism enables the model to reweight diagnostically informative feature maps, including regions corresponding to wing contour, veins, and junctions. Second, residual connections facilitate information flow across the network, helping preserve fine-scale structural details. The resulting latent vector provides a compressed morphological descriptor for each specimen, but it should be interpreted as an image-derived representation rather than as a set of predefined homologous characters.

### Data collection and dataset assembly

We assembled an initial collection of 1003 images of relatively well-preserved forewings from a broad array of extant and extinct insect groups, including Archaeorthoptera (including Caloneurodea, Cnemidolestodea, Titanoptera, and Orthoptera), Dictyoptera (Blattodea), Grylloblattodea, Diptera, Embioptera, Hemiptera, Hymenoptera, Lepidoptera, Mecoptera, Neuroptera, Odonata, Palaeodictyoptera, Paoliida, Psocodea, Phasmatodea, Plecoptera, and Zoraptera. Images were sourced from publications, books, online databases, and museum collections (Fig. 1A). For the subsequent validation and application of HAIKA, we compiled three specialized subdatasets (Fig. 1C). Dataset no. 1 (*n* = 610 species), which includes taxa from all major winged insect groups, was used to assess HAIKA’s performance in taxonomic attribution at a broad phylogenetic scale (Case 1). Dataset no. 2 (*n* = 405 species) focused exclusively on Hymenoptera, covering all subfamilies and major genera, and was also used for taxonomic attribution (Case 1). A relevant subset of this Hymenoptera dataset was used to derive a morphological matrix for phylogenetic tree reconstructions (Case 2; *n* = 61 taxa). Lastly, dataset no. 3 (*n* = 169 species) was restricted to the Hymenoptera species (excluding outgroups) included in the phylogenetic tree by Peters *et al.* (*65*), serving as the topology for macroevolutionary analyses (i.e., ancestral-state reconstructions and rate analyses; Case 3).

### Model architecture of HAIKA

The network is a residual attention autoencoder (Fig. 1B and fig. S4) that maps a standardized grayscale wing image $x\in {\mathbb{R}}^{H\times W}$ (*H* is the height of the image and *W* is the width of the image) to a compact morphology vector $z\in {\mathbb{R}}^{d}$ and reconstructs $\hat{x}$ with a decoder that mirrors the encoder topology shown in Fig. 1B. The encoder consists of a sequence of convolutional stages with residual connections, channel attention, and progressive spatial down sampling. Let $f^{\left(0\right)}=\text{stem}\left(x\right)\in {\mathbb{R}}^{C_{0}\times H_{0}\times W_{0}}$ be an initial feature stack produced by a 3 × 3 convolution followed by batch normalization (BN) (*91*) and a ReLU activation function (*92*) with max pooling, with $H_{0}=\left\lfloor H/2\right\rfloor$ and $W_{0}=\left\lfloor W/2\right\rfloor$, and where $C_{0}$ is a modest width (32 in our experiments). For stages l=1,…,L, features evolve as

$$k^{\left(l\right)}=\phi \left\{\text{BN}\left[{\text{Conv}}_{k_{l},s_{l}}\left(f^{\left(l-1\right)}\right)\right]\right\}$$ (1)

Here, ${\text{Conv}}_{k_{l},s_{l}}$ is a 2D convolution, where $k_{l}$ represents the kernel size (filter width) and $s_{l}$ represents the stride (step size of the filter), ϕ is the ReLU activation function, and spatial sizes were defined as $H_{l}=\left\lfloor \frac{H_{l-1}}{s_{l}}\right\rfloor$, $W_{l}=\left\lfloor \frac{W_{l-1}}{s_{l}}\right\rfloor$, yielding the feature tensor $k^{\left(l\right)}\in {\mathbb{R}}^{C_{l}\times H_{l}\times W_{l}}$. Residual refinement preserves gradient flow and morphology-level detail

$$r^{\left(l\right)}=k^{\left(l\right)}+\Pi^{\left(l\right)}\left(f^{\left(l-1\right)}\right)$$ (2)

with $\Pi^{\left(l\right)}$ is a projection function used to align the dimensions of the feature maps (using a 1 × 1 convolution projection to ${\mathbb{R}}^{C_{l}\times H_{l}\times W_{l}}$) when the channel depths differ. Channel-attention then reweights maps to emphasize sparse, salient structures (e.g., cross-veins and bifurcations in wings). Using a squeeze-excitation form, global average pooling (GAP) (*93*) produces $m^{\left(l\right)}=\text{GAP}\left(r^{\left(l\right)}\right)\in {\mathbb{R}}^{C_{l}}$. The attention gates are

$$\alpha^{\left(l\right)}=\sigma \left[W_{2}^{\left(l\right)}\phi \left(W_{1}^{\left(l\right)}m^{\left(l\right)}\right)\right]$$ (3)

$$f^{\left(l\right)}=\alpha^{\left(l\right)}⊙r^{\left(l\right)}$$ (4)

where $W_{1}^{\left(l\right)}\in {\mathbb{R}}^{\frac{C_{l}}{\rho }\times C_{l}}$ and $W_{2}^{\left(l\right)}\in {\mathbb{R}}^{C_{l}\times \frac{C_{l}}{\rho }}$ are two learnable matrices, ρ is the squeeze-excitation reduction ratio (4 in our experiments), $\alpha^{\left(l\right)}\in {\mathbb{R}}^{C_{l}}$ represents the learned attention weights, ⊙ denotes element-wise multiplication, which scales the features based on their importance, σ is a sigmoid activation function, and $f^{\left(l\right)}\in {\mathbb{R}}^{C_{l}\times H_{l}\times W_{l}}$ denotes the resulting attention-weighted feature maps. A final flatten yields the embedding

$$z=\psi \left(f^{\left(L\right)}\right)$$ (5)

where ψ denotes spatial pooling followed by a linear map, which flattens the feature grid to the latent feature with dimension *d* (64 in our experiments). This vector $z\in {\mathbb{R}}^{d}$ is the compressed, quantitative summary of the wing’s morphology.

The decoder reconstructs the input image from the compact morphology vector **z** by mirroring the encoder’s architecture. This symmetric process requires the latent features to retain sufficient geometric information for reconstruction. The process begins by mapping the latent feature **z** back to a high-dimensional feature space. An initial linear layer followed by reshaping creates a matrix that matches the shape of the encoder’s final feature map

$$g^{\left(L\right)}=\psi^{\prime }\left(z\right)$$ (6)

Here, ψ′ represents the initial projection and reshaping operation.

The network then applies a sequence of up-sampling stages, iterating backward from l=L down to 1. Each stage inverts the corresponding encoder stage’s operations. The feature map from the previous stage, $g^{\left(l\right)}\in {\mathbb{R}}^{C_{L}\times H_{L}\times W_{L}}$, is first spatially enlarged by an up-sampling layer, ${\text{Up}}_{s_{l}}$, which doubles the height and width. This is followed by a convolutional block to refine features

$$h^{\left(l-1\right)}=\phi \left(\text{BN}\left\{{\text{Conv}}_{k_{l},1}^{\prime }\left[{\text{Up}}_{s_{l}}\left(g^{\left(l\right)}\right)\right]\right\}\right)$$ (7)

where ${\text{Conv}}^{\prime }$ is a standard 3 × 3 convolution with a stride of 1 and $h^{\left(l-1\right)}\in {\mathbb{R}}^{C_{l-1}\times H_{l-1}\times W_{l-1}}$ is the refined features.

Just like in the encoder, these stages include residual connections and a channel-attention mechanism to preserve fine anatomical detail. The residual feature map is calculated as

$$r^{\prime \left(l-1\right)}=h^{\left(l-1\right)}+\Pi^{\prime \left(l-1\right)}\left[{\text{Up}}_{s_{l}}\left(g^{\left(l\right)}\right)\right]$$ (8)

where $\Pi^{\prime \left(l-1\right)}$ is a projection (a 1 × 1 convolution) used to match channel dimensions. The channel-attention gates $\alpha^{\prime \left(l-1\right)}$ are then computed and applied multiplicatively

$$\alpha^{\prime \left(l-1\right)}=\sigma \left\{W_{2\left(l-1\right)}^{\prime }\phi \left[W_{1\left(l-1\right)}^{\prime }\text{GAP}\left(r^{\prime \left(l-1\right)}\right)\right]\right\}$$ (9)

The output of the attention block, which serves as the input for the next stage, is

$$g^{\left(l-1\right)}=\alpha^{\prime \left(l-1\right)}⊙r^{\prime \left(l-1\right)}$$ (10)

After the final decoder stage produces the feature map $g^{\left(0\right)}\in {\mathbb{R}}^{C_{0}\times H_{0}\times W_{0}}$, a final 3 × 3 convolutional layer with BN and ReLU projects this matrix back to a single-channel grayscale image, yielding the reconstructed output $\hat{x}\in {\mathbb{R}}^{H\times W}$.

The reconstruction loss $L_{\text{rec}}$ supervises the training process implicitly by

$$L_{\text{rec}}=∥x-\hat{x}{∥}_{2}^{2}$$ (11)

Where $∥\cdot {∥}_{2}^{2}$ represents the squared $L_{2}$ Norm. This term measures the aggregate pixel-wise error, defined as the sum of squared differences between the original input x and the reconstructed $\hat{x}$.

The model was trained on grayscale wing images resized to 128 × 128 and normalized to [0, 1], using a 90/10 train-validation split. The architecture comprises a three-layer encoder and a mirrored decoder, each with residual blocks augmented by channel attention. Training used Adam optimizer (*94*) (learning rate = 10^−3^, batch size = 16) for up to 50 epochs with early stopping on validation loss. The objective combined reconstruction MSE (*95*) with $L_{2}$ regularization (*96*) (weight decay λ = 10^−5^). Convolutions used 3 × 3 kernels; down-/up-sampling used 2 × 2 pooling/up-sampling; the latent dimension was 64. All experiments were run on an NVIDIA RTX A5000 GPU; the entire training process took ∼10 min to complete. For the notation and mathematical symbols used in the model architecture, refer to table S1.

#### Extension of the design to 3D data

For volumetric inputs $x\in {\mathbb{R}}^{D\times H\times W}$, every 2D operator is replaced by its 3D counterpart while preserving widths, depth, residual topology, attention placement, and the loss. Encoder features become $f^{\left(l\right)}\in {\mathbb{R}}^{C_{l}\times D_{l}\times H_{l}\times W_{l}}$ with $D_{l}=\left\lfloor D_{l-1}/s_{l}\right\rfloor$; channel-attention squeezes across (D,H,W) so $m^{\left(l\right)}\in {\mathbb{R}}^{C_{l}}$ and $\alpha^{\left(l\right)}\in {\mathbb{R}}^{C_{l}}$ are unchanged in shape; the latent remains $z\in {\mathbb{R}}^{d}$. The mirrored decoder outputs $\hat{x}\in {\mathbb{R}}^{D\times H\times W}$. On simulated volumetric datasets that mimic layered venation and thickness variation, this drop-in 3D instantiation maintains high reconstruction fidelity and produces embeddings that separate morphological regimes comparably to the 2D case, indicating that the residual-attention inductive bias and reconstruction constraint transfer effectively to the volumetric setting. For the 3D capability demonstration, we voxelized 1000 objects (five categories) from ModelNet10 (*97*) via TensorFlow Datasets and a primate 3D dataset from MorphoSource [six categories, total of 331 volumetric scans; (*98*)], and then, after training, analyzed the embeddings for dimensionality reduction and visualization. The results are shown in figs. S5 and S6.

### Model evaluation

To rigorously assess the performance of HAIKA, we designed an evaluation strategy combining quantitative internal clustering metrics with qualitative external interpretability checks. This approach measured the structure of the learned morphological features and evaluated whether visually emphasized regions coincided with anatomically informative wing regions. The evaluation was conducted on the entire insect wing dataset, comparing HAIKA against 14 fine-tuned baseline networks and four conventional morphology descriptors as complementary, non–deep-learning benchmarks.

To probe the latent features of the wing dataset learned by each model, we analyzed three complementary unsupervised pipelines. We first applied *k*-means (*99*) directly in the original feature space to obtain a baseline measure of linear separability. Next, PCA (*100*) was used to retain the global variance structure before agglomerative clustering, a setting that reveals broad wing-shape trends. Last, *t*-SNE (*101*) was used to unfold nonlinear manifolds; the resulting latent features were again subjected to agglomerative clustering, allowing for the fine examination of venation topology. Using all three pipelines in tandem reduces the likelihood of favoring any single clustering assumption and therefore provides a more balanced view of model behavior.

Cluster quality within each pipeline was quantified with three established internal indices. The SC (*102*) is defined as

$$\text{SC}=\frac{1}{n}\sum_{i=1}^{n}\frac{b\left(i\right)-a\left(i\right)}{\text{max}\left\{a\left(i\right),b\left(i\right)\right\}}$$ (12)

where *n* is the number of samples, *a*(i) is the mean distance from sample *i* to all other points in its own cluster, and *b*(i) is the mean distance from *i* to points in the nearest neighboring cluster. A higher SC indicates tighter and better-separated clusters.

The CH index (*103*) is written as

$$\text{CH}=\frac{\text{tr}\left(B\right)/\left(k-1\right)}{\text{tr}\left(W\right)/\left(n-k\right)}$$ (13)

where *k* is the number of clusters, tr(B) denotes the trace of the between-cluster scatter matrix **B**, and tr(W) is the trace of the within-cluster scatter matrix **W**. A larger CH value reflects a greater ratio of between-cluster dispersion to within-cluster dispersion and therefore superior cluster structure.

Last, the DB index (*104*) is expressed as

$$\text{DB}=\frac{1}{k}\sum_{i=1}^{k}\underset{j\neq i}{\text{max}}\;\frac{s_{i}+s_{j}}{d\left(c_{i},c_{j}\right)}$$ (14)

where $s_{i}$ is the average distance of points in cluster *i* to its centroid $c_{i}$, $d\left(c_{i},c_{j}\right)$ is the Euclidean distance between centroids *i* and *j*, and *k* is the number of clusters. Lower DB values correspond to clusters that are simultaneously compact and well separated from one another.

The 14 reference networks were selected to represent the diversity of major neural network design philosophies (nine supervised convolutional neural networks and five self-supervised neural networks), ensuring a robust comparison against established architectures (Fig. 2A). These nine supervised baselines included plain convolutional networks VGG16 (*105*), residual networks ResNet50 (*106*), densely connected networks DenseNet121 (*107*), models using depth-wise separable convolutions Xception (*108*), multiscale architectures with and without residuals InceptionV3 (*109*) and Inception-ResNet-V2 (*110*), a compound-scaled model EfficientNet-B0 (*111*), and lightweight mobile-oriented designs MobileNetV2 (*112*) and NASNetMobile (*113*). All supervised networks are pretrained on ImageNet and fine-tuned using the insect-wing images. Five self-supervised baselines included asymmetric self-distillation framework BYOL (*114*), simple contrastive learning framework SimCLR (*115*), foundational feature learning network AutoEncoder (*88*), momentum-based contrastive learning method MoCo (*116*), and probabilistic generative model VAE (*89*). In addition to the 14 deep-learning baselines, we benchmarked four conventional descriptors computed from the same standardized wing images. Although these descriptors are useful reference points, they each capture only a limited aspect of wing morphology, such as outline shape, local gradients, pseudo-landmark (pseudo-LM) distributions, or simplified venation topology. Wing outline was summarized using normalized elliptic Fourier descriptors (EFDs), local contour and venation information using histogram of oriented gradients (HOG), landmark-like information using automatically detected corner points (pseudo-LM), and coarse venation topology using a skeleton-based venation graph descriptor.

As shown in Fig. 2B, we also performed external visual evaluation by visualizing the regions each network attended to during inference. Grad-CAM++ saliency maps (*117*) were generated for the CNN baselines that do not contain attention mechanisms, and attention rollout (*118*) was applied to HAIKA and CNN baselines with attention mechanisms.

All models were fine-tuned with an identical schedule (AdamW optimizer, cosine-annealed learning-rate decay from 1 × 10^−4^ to 1 × 10^−6^ over 50 epochs, batch size 32) before metric computation to ensure comparability. To facilitate comparison across architectures, all methods were evaluated using latent representations of the same dimensionality. For each baseline, the representation used for downstream analyses was extracted from the final feature embedding produced before the task-specific prediction layer. Other hyperparameters were tuned individually for each model on the basis of validation loss, rather than being imposed uniformly across all architectures, in order to accommodate model-specific optimization requirements. The resulting embeddings were then subjected to the same downstream evaluation pipeline and internal validity metrics.

Model training and evaluation were conducted in Python 3.11 (www.python.org/) with PyTorch 2.1 (https://pytorch.org) + CUDA 11.8 (https://developer.nvidia.com/cuda-toolkit), NumPy (https://github.com/numpy/numpy), Pandas (https://pandas.pydata.org), Matplotlib (https://github.com/matplotlib/matplotlib), and TensorFlow (https://github.com/tensorflow/tensorboard). Clustering and index calculations used scikit-learn 1.3 (https://scikit-learn.org/stable); *t*-SNE relied on openTSNE 1.0 (https://opentsne.readthedocs.io/en/stable/); visualizations were produced with Captum (https://github.com/meta-pytorch/captum), grad-cam (https://github.com/jacobgil/pytorch-grad-cam) and Matplotlib (https://github.com/matplotlib/matplotlib) / Seaborn (https://seaborn.pydata.org/).

### Morphological matrix extraction

To ensure consistency, standardized orthogonal views were analyzed to generate high-quality binary (black-and-white) line drawings (Fig. 1A). All illustrations underwent manual verification to confirm morphological fidelity before subsequent processing. The preparation workflow began by setting each specimen’s wing orientation parallel to the long axis of a lateral B5-format canvas in Adobe Illustrator (version CC 2023). Wing illustrations were generated using two distinct protocols. For specimens with existing schematic diagrams in the literature, we performed automated bitmap tracing using Inkscape (v1.2.1), followed by manual refinement to eliminate extraneous annotations. For specimens lacking preexisting illustrations, standardized vector line drawings were created de novo through manual digitization following established entomological illustration protocols.

All resulting illustrations specifically emphasized the primary venation patterns while deliberately excluding highly variable traits (at this large taxonomic scale) such as chromatic and spatial distribution characteristics. Processed images were then uniformly converted to bitmap format (300 dpi, grayscale). The preprocessed wing images served as input for the HAIKA model, which automatically extracted a 64-dimensional continuous feature matrix encapsulating essential morphological characteristics through hierarchical feature abstraction (Fig. 1B). The dimension of the feature matrix is adjustable to adapt to the complexity of the feature information. We used HAIKA to extract continuous trait matrices for all three previously assembled subdatasets.

### Taxonomic attribution (Case 1)

In Case 1, we assessed HAIKA’s performance in attributing the correct taxonomic attribution to wing images across two different taxonomic scales: the broad insect dataset no. 1 spanning 20 higher groups of insects, and the Hymenoptera-focused dataset no. 2. The evaluation procedure comprised four sequential steps designed to validate the morphological signals statistically and quantitatively. First, we carried out PCA to reconstruct the latent wing morphological space (Fig. 1C). We evaluated the relative weights of the first 10 PCs through eigenvalue decomposition and used established taxonomic labels to display the distribution of species and the centroid of each group for visualization purposes. The labels used were Acercaria, Holometabola, Palaeoptera, and Polyneoptera for the first dataset, and Aculeata, parasitoid groups (A: Ichneumonoidea + Ceraphronoidea; B: Evanioidea + Stephanoidea + Megalyroidea + Trigonaloidea), and Symphyta for the Hymenoptera dataset, based on phylogenies from Nel *et al.* (*119*), Ronquist *et al.* (*34*), Misof *et al.* (*120*), Peters *et al.* (*65*), Wipfler *et al.* (*121*), and Blaimer *et al.* (*58*). These labels were not used during the feature extraction process, as HAIKA is an unsupervised model. Second, we performed a PERMANOVA to statistically validate the observed taxonomic clustering within the morphospace. This analysis was based on a Euclidean distance matrix derived from the HAIKA-extracted feature matrix. We conducted pairwise comparisons between all predefined taxonomic groups to test the null hypothesis of no significant difference between the groups in multivariate space. *P* values were derived from 9999 permutations to ensure robustness. Homogeneity of multivariate dispersion was assessed using PERMDISP/betadisper (*122*). Third, the final quantitative measure of classification accuracy was obtained using supervised LDA. The latent representations produced by HAIKA were treated as predictor variables, with the known taxonomic identities used as target classes. We used two complementary metrics of classification accuracy: resubstitution accuracy (applying the trained LDA model back onto the original training dataset) and the more robust cross-validated accuracy obtained via a leave-one-out (jackknifing) protocol. These two metrics together provide a reliable measure of the biological and taxonomic information encoded by the HAIKA framework. Fourth, we conducted an ablation experiment, removing the residual connection and attention mechanism from HAIKA, to quantify the effectiveness of HAIKA’s architectural innovations. LDA was repeated, as in step 3, after the latent matrix was extracted with the residual connections and attention mechanism removed from the model. All statistical analyses were performed using the PAST software (*123*).

### Phylogenetic tree reconstruction (Case 2)

To test the contribution of the continuous morphological matrix extracted using HAIKA to phylogenetic reconstructions (Fig. 1C), we assembled three morphological character matrices of Hymenoptera (subset of dataset no. 2, *n* = 61 taxa). These included a pre-established discrete morphological matrix of extant species (343 characters) derived from the one in Ronquist *et al.* (*34*), a 64-dimensional continuous character matrix extracted from wings using HAIKA, and a combined matrix incorporating both sources. Because the latent dimensions are jointly learned and are not guaranteed to be independent, we limited their aggregate influence in the combined matrix. Specifically, the 64 continuous dimensions were collectively assigned a total weight equivalent to the 13 wing-related discrete characters in Ronquist *et al.* (*34*), rather than being counted as 64 independent character units. To assess sensitivity to overlap between data sources, we also analyzed a combined matrix from which those 13 discrete wing characters were removed; its topology showed no meaningful change in node recovery relative to the full combined matrix, although bootstrap support values were moderately reduced.

Phylogenetic analyses were conducted using parsimony in TNT 1.6 software (*124*). All discrete characters were treated as unordered with equal transformation costs. We used an iterative search protocol combining Wagner tree initialization, tree bisection and reconnection (TBR) branch swapping, sectorial searches, Ratchet perturbation, and tree fusion algorithms. This process was repeated for 100 replicates until topological stability was achieved. An austropyridine (Plecoptera) was used as an outgroup, replacing the original outgroups from Ronquist *et al.* (*34*), which were not sampled in our datasets.

Given the inherent challenge of achieving high resolution in morphology-based phylogenies, and because our primary aim was to compare matrix performance, we analyzed each matrix with and without six predefined topological constraints (monophyly of Pamphilioidea, Siricoidea, Apocrita, Xyelidae, Tenthredinidae, and Vespina), following Ronquist *et al.* [see their data availability in (*34*)]. Final optimization involved exhaustive TBR swapping on the retained shortest trees, collapse of zero-length branches, and assessment of branch robustness through bootstrap resampling (1000 pseudoreplicates). We reconstructed strict consensus trees and compared our results against a reference phylogenetic tree [the one found under a nonclock model in Ronquist *et al*.; see figure 3 in (*34*)]. The final results were edited and enhanced using Adobe Illustrator (version CC 2023).

### Phylogenetic signal, ancestral state reconstruction, and trait evolution (Case 3)

In Case 3, we leveraged HAIKA’s derived characters to inform our understanding of wing evolution in Hymenoptera, building on the assumption that morphological evolution is driven by functional demands, ecological transitions, and key innovations (*125*). We specifically investigated the concomitant evolution of wing shape and venation, and the temporal correlation between major morphological changes and defined evolutionary events (e.g., predation, parasitoidism, and stinger and wasp-waist evolution).

To counter the common “black box” problem in AI models (*126*) and ensure the biological clarity of HAIKA’s output, we designed a strategy to relate the abstract latent characters to five quantifiable classical morphological characters of established systematic and functional significance in Hymenoptera. We selected two characters related to wing shape and flight performance: AR and SMC. We also chose three characters related to venation complexity: number of closed cells, pterostigma state, and distal extension of wing veins (*26*, *127*–*129*).

These classical characters were quantified via two approaches. Wing shape parameters (AR and SMC) were computed using a pipeline in which the wing contour was isolated and a mask was generated (detailed in the “The quantification method of wing shape parameters” section in the Supplementary Materials). Total wing area (*S*) and wing span (*b*) were then computed (*29*, *31*), allowing us to derive AR (AR = *b*^2^ / *S*) and SMC (SMC = *S* / *b*). For venation, the number of closed cells was used as a direct count-based proxy for venational complexity (*128*). The number of closed cells was determined by a count of all fully enclosed areas bounded by sclerotized veins. Pterostigma state, an important structure in flight stability, was coded on a four-point scale representing its development: absent/vestigial (0), small/reduced (1), well-developed (2), and exceptionally large/broad (3). Last, distal extension of wing veins, an inverse proxy of vein reduction, was also coded on a four-point scale from absent (0), not more than half (1), not reaching the margin (2), and complete (i.e., reaching the margin) (3).

The HAIKA-derived continuous characters were first simplified via a PCA. We verified that PC1 and PC2 sufficiently represented the bulk of the morphological variation. We then performed a multivariate multiple linear regression using PC1 and PC2 as dependent variables and the five morphological characters as independent predictors. To mitigate multicollinearity between AR and SMC, two separate regression models were executed. Significant correlations were used to biologically interpret the results of the subsequent comparative analyses.

All phylogenetic comparative analyses were carried out on the PC1 and PC2 scores using the time-calibrated Hymenoptera phylogeny of Peters *et al.* (*65*). We quantified the phylogenetic signal of PC1 and PC2 using two metrics: Blomberg’s *K* (*130*) and Pagel’s λ (*131*). We then fitted three time-dependent macroevolutionary models to the PC scores: Brownian Motion (BM), Ornstein-Uhlenbeck (OU), and Early Burst (EB). Model fit was evaluated using the Akaike Information Criterion corrected (AICc). To test whether evolutionary rates differed significantly between PC1 and PC2, we extracted the rate parameter (σ^2^) from the best-fitting evolutionary model and calculated the ratio of the rates of PC1 and PC2. This observed ratio was compared to a distribution with expected values from 1000 bootstrap simulations under the null hypothesis of equal rates.

Lastly, we performed ASRs for PC1 and PC2 using a maximum-likelihood framework [fastAnc of phytools in R; (*132*)]. Ancestral state values were mapped along the branches of the phylogeny using the contMap function. We quantified major above-average changes by defining Δ at a given node as the absolute difference between its ASR value and that of its immediate ancestral node. It was not treated as an outlier test, formal shift-detection model, or test of ecological causality. We focused our comparison of these Δ values with known ecological and behavioral shifts in Hymenoptera at the deepest nodes (i.e., mostly above the superfamily level) to avoid sampling bias associated with shallower nodes.

All analyses were conducted in the R environment [v4.4.1; (*133*)], using the following packages: ape (*134*), phytools (*132*), geiger (*135*), and ggplot2 (*136*).

## Supplementary Materials

### The PDF file includes:

Supplementary Text Figs. S1 to S8 Table S1 Legends for tables S2 to S11 References
