# Supplementary material for: Deep learning reveals ecological decoupling and rate heterogeneity in Hymenoptera wing evolution
Source: Sci Adv. 2026 Oct 14;12(42):eaef0605. doi: 10.1126/sciadv.aef0605 (PMC13665928; doi:10.1126/sciadv.aef0605)
Supplement: Supplementary file 1 [file sciadv.aef0605_sm.pdf]

Science Advances
AAAS

Supplementary Materials for

Deep learning reveals ecological decoupling and rate heterogeneity in Hymenoptera wing evolution

Ancheng Peng *et al.*

Corresponding author: Shihan Guan, shihan.guan@univ-rennes.fr

Sci. Adv. **12**, eaef0605 (2026)
DOI: 10.1126/sciadv.aef0605

The PDF file includes:

Supplementary Text
Figs. S1 to S8
Table S1
Legends for tables S2 to S11
References

Other Supplementary Material for this manuscript includes the following:

Tables S2 to S11

Supplementary Text

The quantification method of wing shape parameters

The wing shape parameters (AR and SMC) were quantified using a multi-step computational pipeline designed for reproducible feature extraction. First, the wing contour was isolated from each image using the Segment Anything Model [SAM, (*87*)] with a Vision Transformer backbone [ViT-B, (*137*)]. For comprehensive segmentation, we employed an automatic mask-generation procedure that identifies and delineates visible structures in the image without manual prompting. The generation parameters were configured to be inclusive, with a predicted Intersection over Union (IoU) threshold of 0.8 and a stability-score threshold of 0.9, so that fine-scale features were retained. From the resulting candidate masks, the one encompassing the largest pixel area, corresponding to the complete wing silhouette, was selected for subsequent analysis.

Following extraction of the binary wing mask, a custom computational routine was used to calculate the morphological parameters. The total wing area (S) was computed by summing the non-zero pixels in the selected mask. Wing span (b) was determined by fitting a minimum-area bounding rectangle to the wing contour; the length of the major axis of this rectangle was taken as the span. This workflow provides a consistent high-throughput approach for quantifying wing morphology across the dataset.

Region-level concordance of HAIKA attention

To avoid treating attention maps as causal explanations, we used them only as qualitative plausibility checks and added a region-level concordance assessment. In 210 Hymenoptera images, the dominant HAIKA attention region included the expert-recognized midwing region (Fig. S8) in 203 cases (96.7%). This result indicates that the attention pattern usually overlaps with an anatomically informative wing region, but it does not establish formal homology or causal interpretability.

Repeatability and embedding stability

To assess stability across model initialization, HAIKA training and evaluation were repeated across five independent random seeds. The resulting clustering metrics remained stable across PCA, t-SNE, and k-means evaluations (table S9). Cross-seed comparisons of embedding geometry and clade-cohesion structure also showed high consistency (table S10). These analyses reduce the likelihood that the reported benchmark results reflect a single favorable initialization.

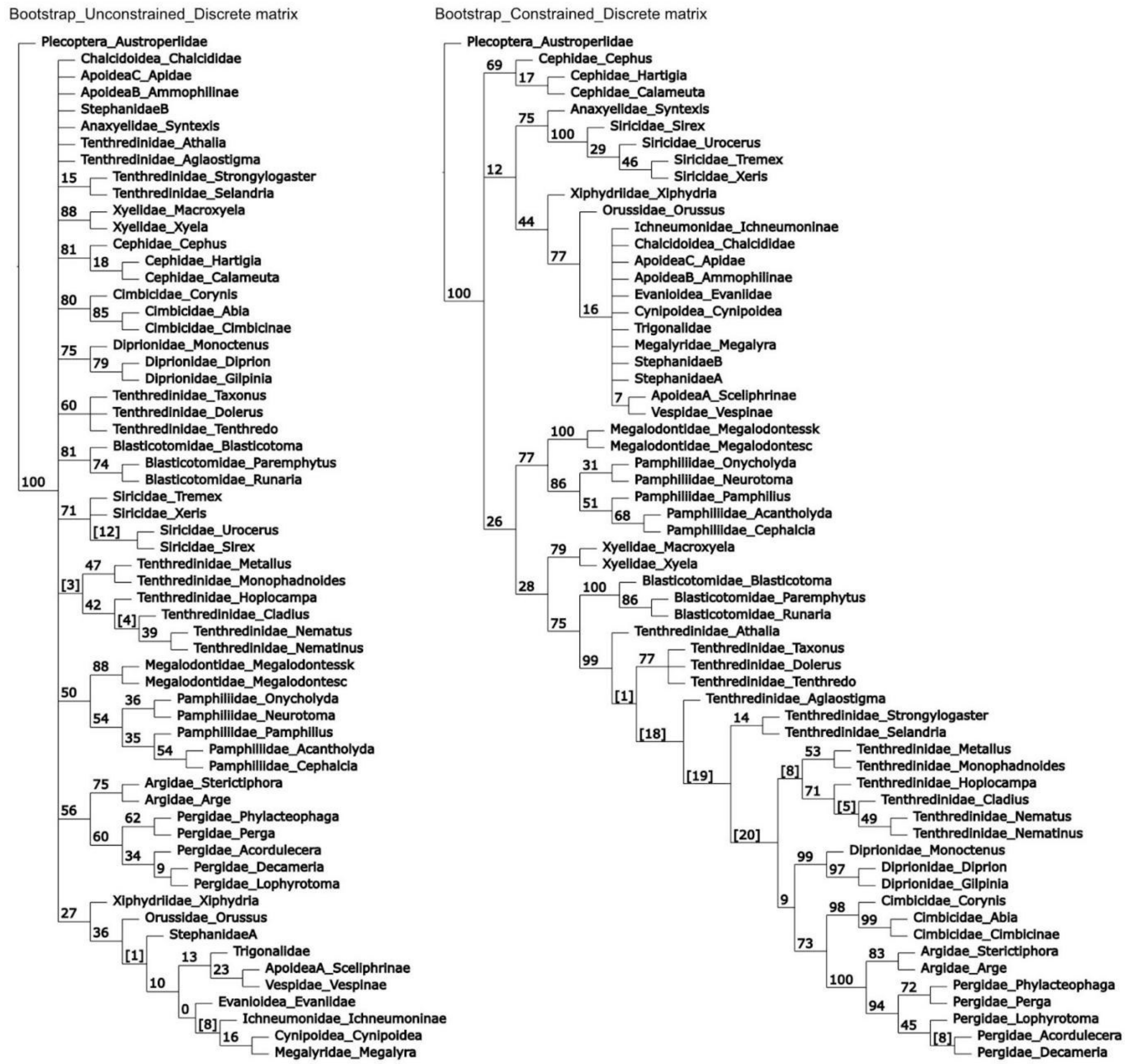

Fig. S1. Phylogenetic analyses and nodal support for the discrete matrix (Case 2) using TNT (V1.6). Strict consensus trees obtained from the unconstrained analysis (left) and the constrained analysis (right) under equal weighting. Due to the high level of homoplasy in the discrete dataset, both searches reached the memory buffer limit, retaining > 10,000 equiparsimonious trees. The unconstrained analysis yielded a tree length of 1245 steps (CI = 0.355; RI = 0.723), whereas the constrained analysis resulted in a tree length of 1280 steps (CI = 0.345, RI = 0.710). Bootstrap resampling values are indicated at the corresponding nodes.

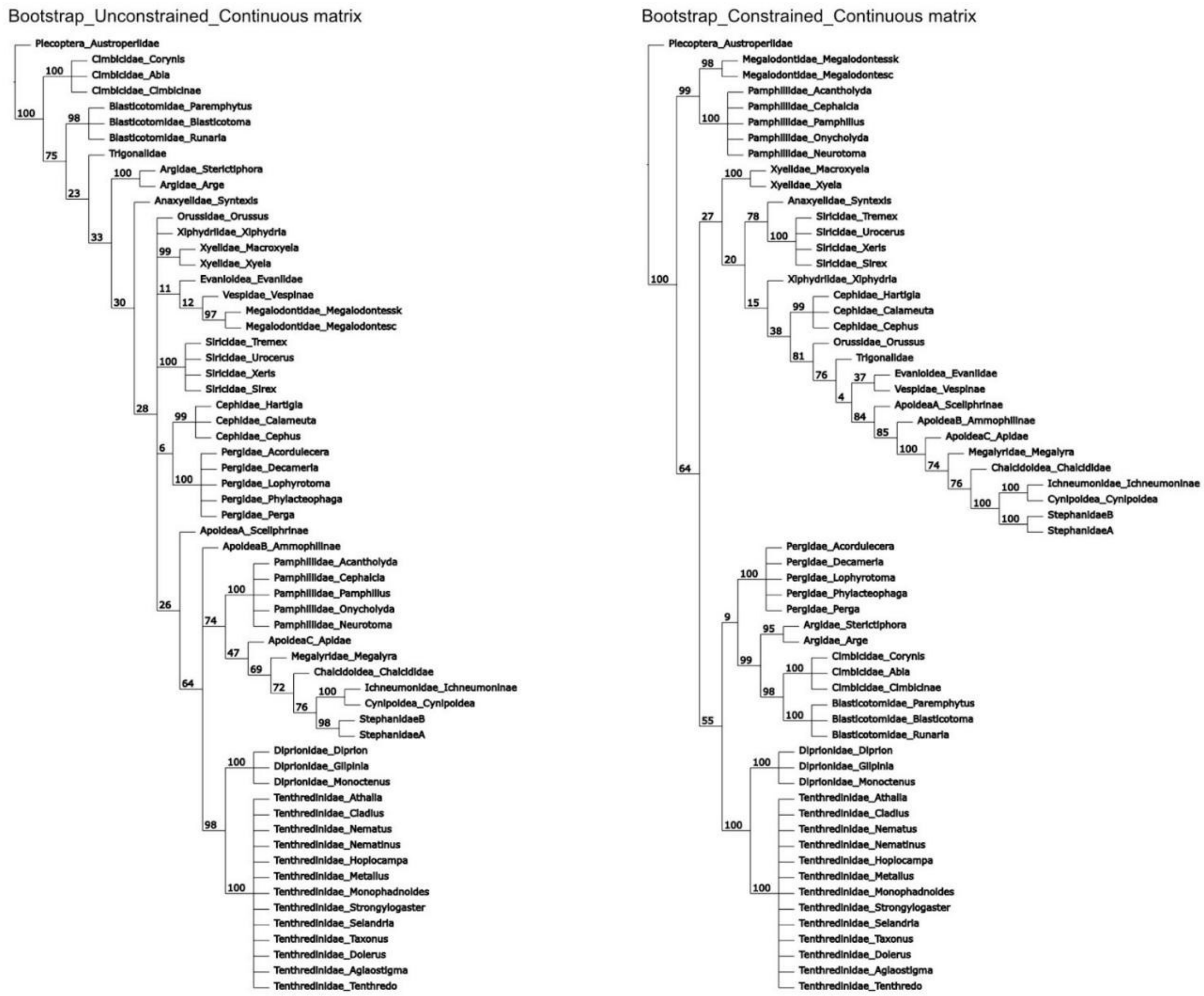

Fig. S2. Phylogenetic analyses and resampling support for the continuous matrix (Case 2) in TNT (V1.6). Strict consensus trees resulting from the unconstrained analysis (left) and the constrained analysis (right) under equal weighting. The unconstrained search retrieved five equiparsimonious trees (length = 92, CI = 0.921, RI = 0.880), whereas the constrained search yielded a single most parsimonious tree (length = 96, CI = 0.883, RI = 0.813). Bootstrap support values are indicated at the corresponding nodes.

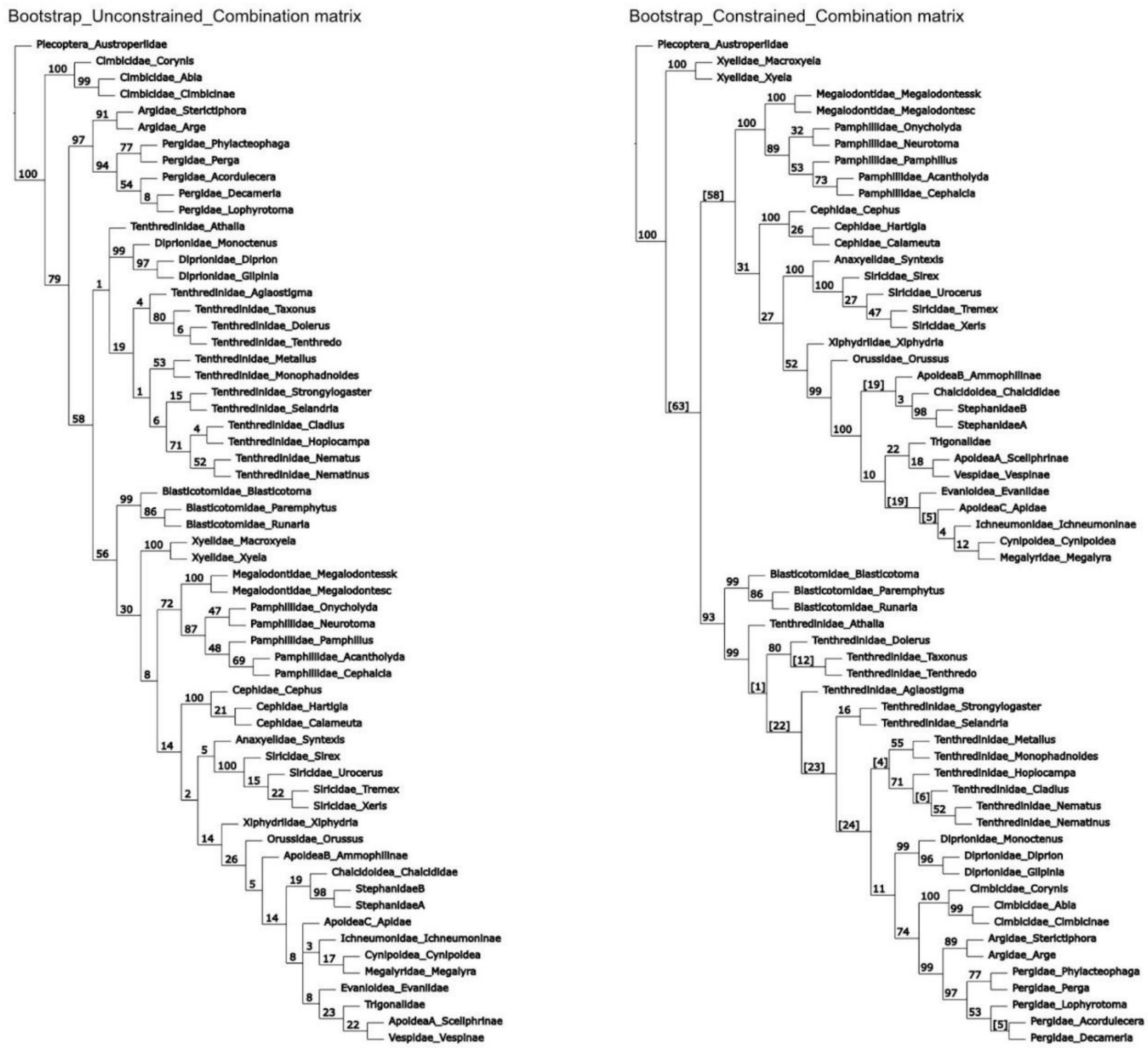

Fig. S3. Phylogenetic analyses and resampling support for the combination matrix (Case 2) under equal weighting in TNT (V1.6). The strict consensus trees are shown for the unconstrained analysis (left) and the constrained analysis (right). The unconstrained search yielded 1302 equiparsimonious trees (length = 1345, CI = 0.391, RI = 0.723), while the constrained search resulted in 762 equiparsimonious trees with a marginally higher tree length (length = 1349, CI = 0.390, RI = 0.722). Bootstrap support values are indicated at the corresponding nodes.

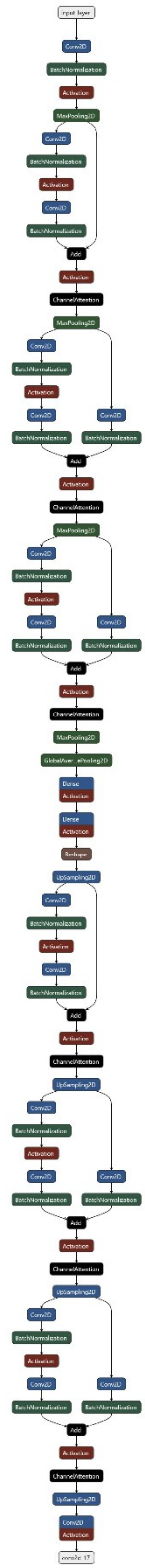

Fig. S4. A diagram of HAIKA's complete model architecture.

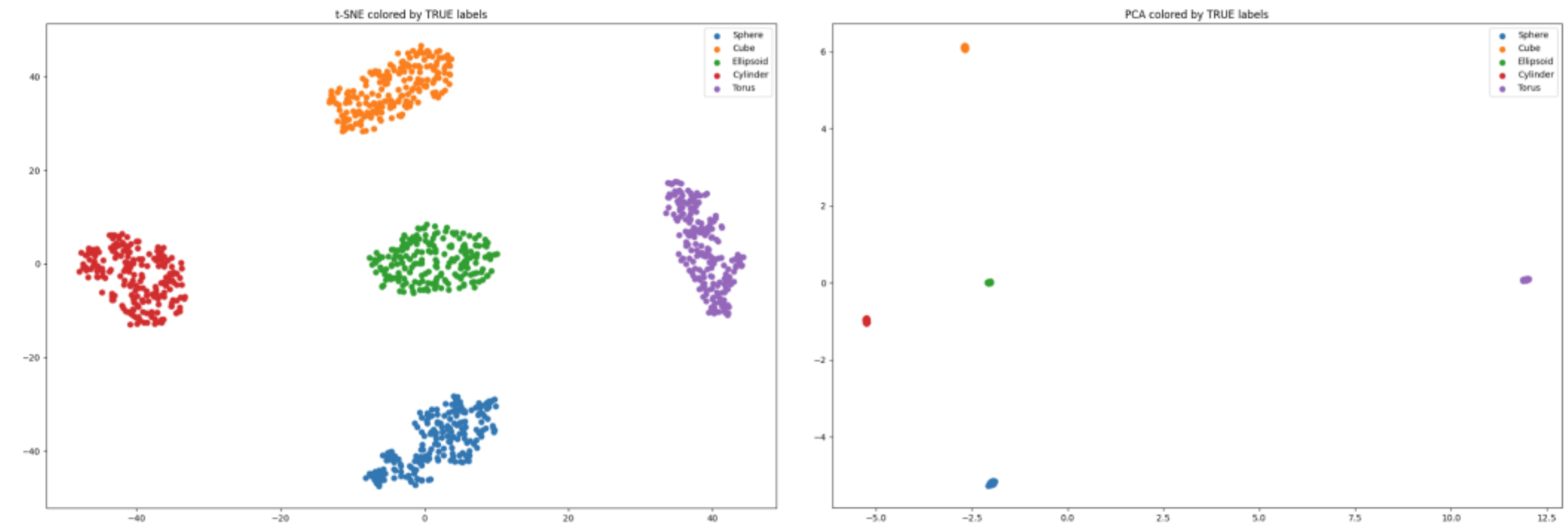

Fig. S5. Proof-of-concept t-SNE and PCA visualization of the standard 3D volume dataset. High-dimensional features from five 3D volumetric classes were reduced to a two-dimensional embedding space using t-distributed Stochastic Neighbor Embedding (t-SNE) and PCA. HAIKA-derived morphological descriptors were used as input features. Points are colored according to class labels (Sphere, Cube, Ellipsoid, Cylinder, Torus). The separation among classes illustrates the architectural flexibility of HAIKA for volumetric inputs, but this analysis is treated as a proof of concept rather than as a central empirical result. The raw embedded matrix is provided in the online repository cited in Data and Materials Availability.

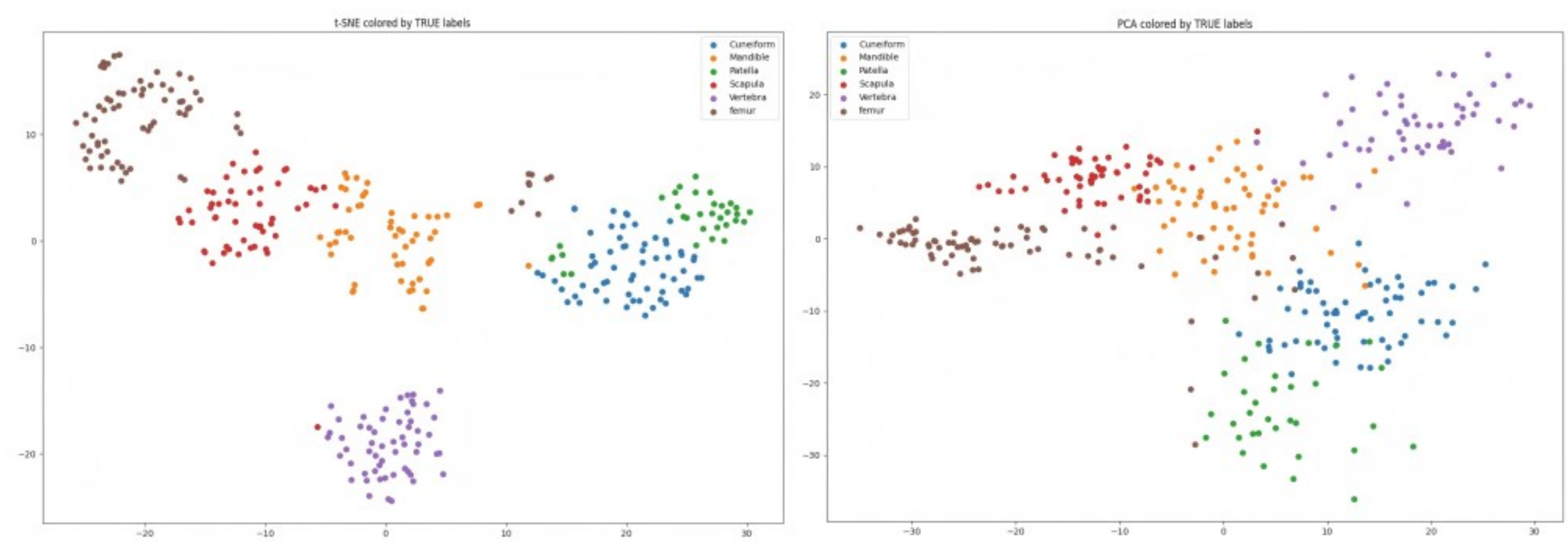

Fig. S6. Proof-of-concept t-SNE and PCA visualization of the primate 3D dataset. High-dimensional features from six volumetric classes were reduced to a two-dimensional embedding space using t-distributed Stochastic Neighbor Embedding (t-SNE) and PCA. HAIKA-derived morphological descriptors were used as input features. Points are colored according to class labels (Cuneiform, Mandible, Patella, Scapula, Vertebra, and Femur). The raw volumetric data were downloaded from MorphoSource [ID: 000656244, (*98*)], and the embedded matrix is provided in the online repository cited in Data and Materials Availability. This analysis is presented as a proof of concept for 3D data handling.

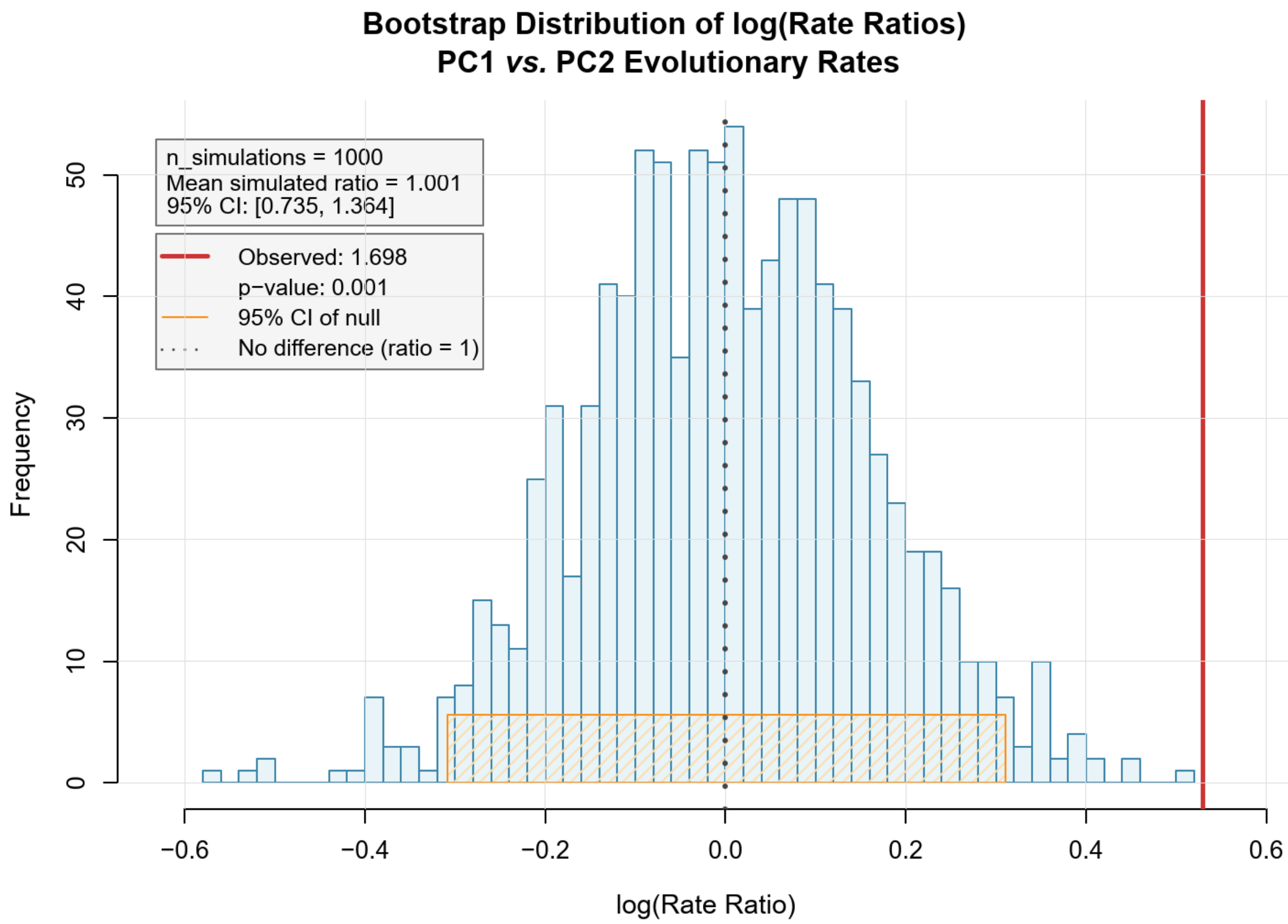

Fig. S7. Comparison of evolutionary rates between morphological trait axes PC1 and PC2. The histogram displays the bootstrap distribution of the log-transformed ratio of evolutionary rates for PC1 versus PC2 based on 1000 simulations. This distribution represents the null hypothesis that the rates are equal (log-ratio = 0). The solid red vertical line indicates the observed log (rate ratio), corresponding to an untransformed ratio of 1.698. The 95% confidence interval of the null distribution is [0.735, 1.364]. The observed ratio falls outside this interval (P = 0.001), indicating that the estimated evolutionary rate of PC1 is greater than that of PC2 under the fitted model.

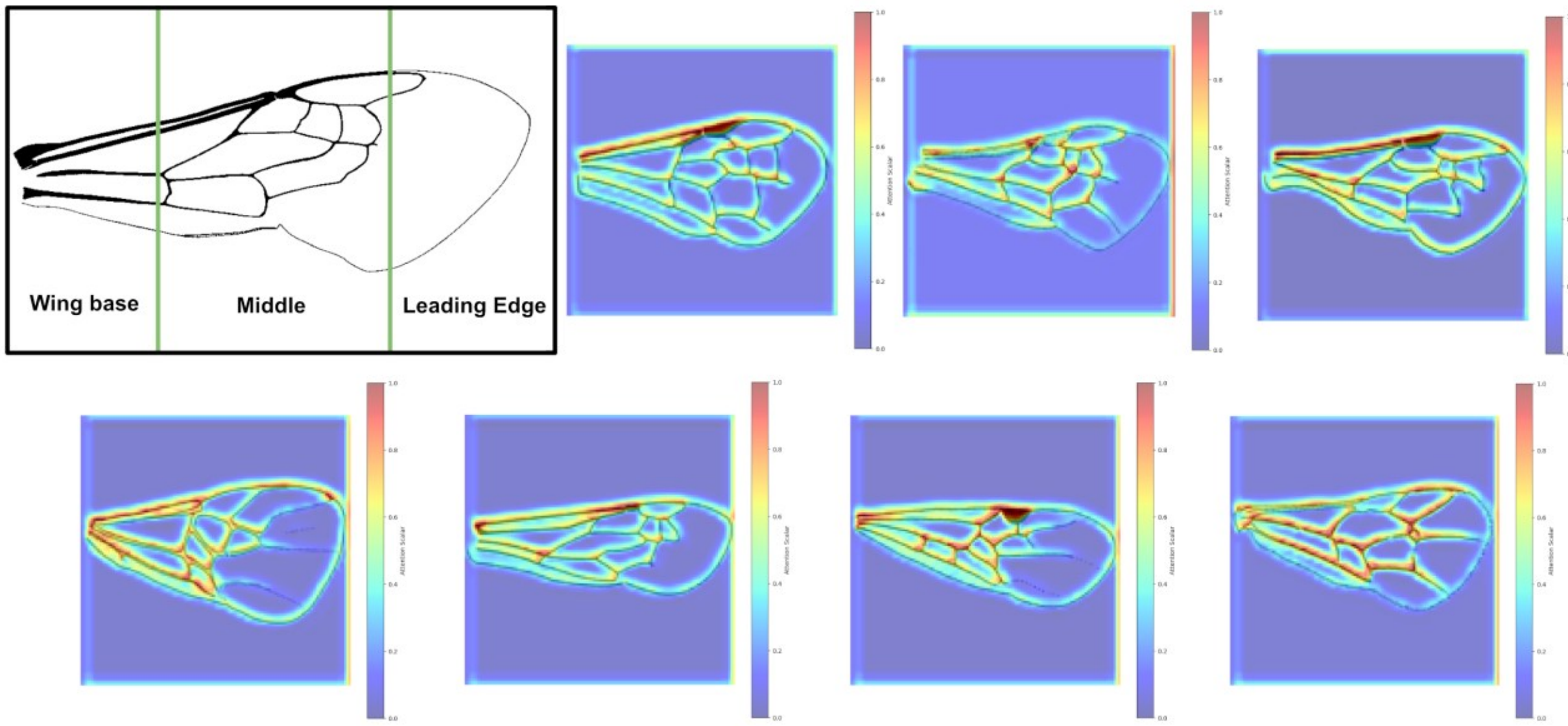

Fig. S8. Comparison between expert-recognised informative regions and HAIKA attention distributions in hymenopteran wings. The 'middle' region in the top left image shows the information-rich region judged by the expert [from Goulet & Huber, 1993's (*127*) key to Hymenoptera families], while the rest shows randomly selected attention maps from HAIKA (the complete dataset is available in the online Zenodo repository).

Table S1. Notation and mathematical symbols used in the HAIKA model architecture. This table summarizes the variables and operations governing the encoder-decoder transformations and channel-attention mechanism described in Equations 1 to 11.

Symbol	Definition	Description
Data		
x	Input Image	The standardized grayscale input image representing the specimen.
H, W, D	Spatial Dimensions	The height (H), width (W), and depth (D, for 3D data) of the input or feature maps.
z	Latent Feature	The compressed morphological feature vector (dimension $d = 64$) extracted by the encoder.
Cl	Channel Depth	The number of feature channels (filters) at layer l.
Network Operations		
$f(l)$	Feature Map	The intermediate feature representation output by the l-th stage of the encoder.
Conv	Convolution	A filtering operation that extracts local patterns (e.g., edges, veins) from the image or features.
BN	Batch Normalization	A technique used to normalize layer inputs, stabilizing the training process.

$\phi(\cdot)$	ReLU Activation	Rectified Linear Unit function; introduces non-linearity by setting negative values to zero.
(l)	Residual Projection	A linear mapping (1×1 convolution) used to align feature dimensions within residual connections.
Ups	Up-sampling	An operation in the decoder that increases the spatial resolution of feature maps.
Attention Mechanism		
$r(l)$	Refined Features	Feature maps obtained after the residual connection but before attention weighting is applied.
$m(l)$	Global Average	A vector summarizing the global importance of each feature channel.
(l)	Attention Gates	Learned weights (values between 0 and 1) that highlight biologically relevant regions.
$(\cdot)$	Sigmoid Function	An activation function that squashes values into the range [0,1], used to generate attention probabilities.
$\odot$	Hadamard Product	Element-wise multiplication, used to apply the attention weights to the feature maps.
Loss Function & Optimization		
$W1$, $W2$	Learnable Weights	Matrices of parameters that are updated during training to minimize error.
Lrec	Reconstruction Loss	The objective function quantifies how well the model reconstructs the original image.
$\|\cdot\|22$	Squared $L2$ Norm	The sum of squared differences; used here to calculate the error between the original (x) and reconstructed (x) images.

Supplementary Tables S2 to S11

These tables are provided in the accompanying Excel workbook, with table descriptions included at the top of each worksheet.

Table S2. Benchmark performance of HAIKA, 14 deep-learning baselines, and four conventional descriptors across PCA, t-SNE, and k-means evaluation metrics.

Table S3. Summary of variance explained, phylogenetic signal, best-fit evolutionary model, AICc weight, and evolutionary rate for PC1 and PC2 in Case 3.

Table S4. Ancestral-state reconstruction values for wing trait axes on the time-calibrated Hymenoptera phylogeny of Peters et al. (*65*).

Table S5. Species-level PC scores and node-level ancestral-state reconstruction changes (Δ) for wing trait axes on the phylogeny of Peters et al. (*65*).

Table S6. Clade-level mean PC scores and summary values for five wing-related morphological variables.

Table S7. Multivariate multiple linear regression of PC scores against wing-related characteristics, with Aspect Ratio included as the wing-shape predictor.

Table S8. Multivariate multiple linear regression of PC scores against wing-related characteristics, with Standard Mean Chord included as the wing-shape predictor.

Table S9. Mean and standard deviation of the main clustering metrics from five independent HAIKA runs using five random seeds.

Table S10. Cross-seed stability of embedding geometry and clade-cohesion structure in the HAIKA-derived feature space.

Table S11. PERMDISP tests for dispersion homogeneity associated with the PERMANOVA analyses in the broad insect dataset (#1) and Hymenoptera dataset (#2).

REFERENCES

1. J. J. Wiens, Character analysis in morphological phylogenetics: Problems and solutions. *Syst. Biol.* **50**, 689–699 (2001).

2. J. J. Wiens, The role of morphological data in phylogeny reconstruction. *Syst. Biol.* **53**, 653–661 (2004).

3. G. Giribet, G. D. Edgecombe, The phylogeny and evolutionary history of arthropods. *Curr. Biol.* **29**, R592–R602 (2019).

4. R. A. Pyron, Post-molecular systematics and the future of phylogenetics. *Trends Ecol. Evol.* **30**, 384–389 (2015).

5. D. Grimaldi, M. S. Engel, *Evolution of the Insects* (Cambridge Univ. Press, 2005).

6. T. A. Heath, J. P. Huelsenbeck, T. Stadler, The fossilized birth-death process for coherent calibration of divergence-time estimates. *Proc. Natl. Acad. Sci. U.S.A.* **111**, E2957–E2966 (2014).

7. J. Clavel, G. Escarguel, G. Merceron, mvMORPH: An R package for fitting multivariate evolutionary models to morphometric data. *Methods Ecol. Evol.* **6**, 1311–1319 (2015).

8. T. Schubnel, A. Mazurier, A. Nel, P. Grandcolas, L. Desutter-Grandcolas, F. Legendre, R. Garrouste, Flat does not mean 2D: Using X-ray microtomography to study insect wings in 3D as a model for comparative studies. *Methods Ecol. Evol.* **14**, 2036–2048 (2023).

9. D. M. Boyer, J. Puente, J. T. Gladman, C. Glynn, S. Mukherjee, G. S. Yapuncich, I. Daubechies, A new fully automated approach for aligning and comparing shapes. *Anat. Rec.* **298**, 249–276 (2015).

10. E. Hartop, A. Srivathsan, F. Ronquist, R. Meier, Towards large-scale integrative taxonomy (LIT): Resolving the data conundrum for dark taxa. *Syst. Biol.* **71**, 1404–1422 (2022).

11. M. A. Wills, "Morphological Disparity: A Primer" in *Fossils, Phylogeny, and Form: An Analytical Approach* (Springer, 2001), pp. 55–144.

12. S. Gerber, Use and misuse of discrete character data for morphospace and disparity analyses. *Palaeontology* **62**, 305–319 (2019).

13. K. Karbstein, L. Kösters, L. Hodač, M. Hofmann, E. Hörandl, S. Tomasello, N. D. Wagner, B. C. Emerson, D. C. Albach, S. Scheu, S. Bradler, J. de Vries, I. Irisarri, H. Li, P. Soltis, P. Mäder, J. Wäldchen, Species delimitation 4.0: Integrative taxonomy meets artificial intelligence. *Trends Ecol. Evol.* **39**, 771–784 (2024).

14. R. Hunt, J. L. Reyes-Hernández, J. J. Shaw, A. Solodovnikov, K. S. Pedersen, Integrating deep learning derived morphological traits and molecular data for total-evidence phylogenetics: Lessons from digitized collections. *Syst. Biol.* **74**, 453–468 (2025).

15. S. Braichenko, R. Borges, C. Kosiol, Phylogenetic methods meet deep learning. *Genome Biol. Evol.* **17**, evaf177 (2025).

16. D. Silvestro, C. Pimiento, Emerging uses of artificial intelligence in deep time biodiversity research. *Nat. Rev. Biodivers.* **1**, 671–677 (2025).

17. A. R. Van Dam, L. Štarhová Serbina, Descriptron: Artificial intelligence for automating taxonomic species descriptions with a user-friendly software package. *Syst. Entomol.* **51**, e70005 (2025).

18. T. T. Høye, J. Ärje, K. Bjerge, O. L. P. Hansen, A. Iosifidis, F. Leese, H. M. R. Mann, K. Meissner, C. Melvad, J. Raitoharju, Deep learning and computer vision will transform entomology. *Proc. Natl. Acad. Sci. U.S.A.* **118**, e2002545117 (2021).

19. M. Valan, K. Makonyi, A. Maki, D. Vondráček, F. Ronquist, Automated taxonomic identification of insects with expert-level accuracy using effective feature transfer from convolutional networks. *Syst. Biol.* **68**, 876–895 (2019).

20. M. Hofmann, S. Kiel, L. M. Kösters, J. Wäldchen, P. Mäder, Inferring taxonomic affinities and genetic distances using morphological features extracted from specimen images: A case study with a bivalve data set. *Syst. Biol.* **73**, 920–940 (2024).

21. J. Kukalová-Peck, Origin and evolution of insect wings and their relation to metamorphosis, as documented by the fossil record. *J. Morphol.* **156**, 53–125 (1978).

22. V. Medved, J. H. Marden, H. W. Fescemyer, J. P. Der, J. Liu, N. Mahfooz, A. Popadić, Origin and diversification of wings: Insights from a neopteran insect. *Proc. Natl. Acad. Sci. U.S.A.* **112**, 15946–15951 (2015).

23. L. A. Treidel, K. D. Deem, M. K. Salcedo, M. H. Dickinson, H. S. Bruce, C.-A. Darveau, B. H. Dickerson, O. Ellers, J. R. Glass, C. M. Gordon, J. F. Harrison, T. L. Hedrick, M. G. Johnson, J. E. Lebenzon, J. H. Marden, K. Niitepõld, S. P. Sane, S. Sponberg, S. Talal, C. M. Williams, E. S. Wold, Insect flight: State of the field and future directions. *Integr. Comp. Biol.* **64**, 533–555 (2024).

24. J. Białkowski, J. Gohli, R. Rossa, A. Ziemiakowicz, J. Goczął, Evolutionary constraints shape the diversity of microinsects' wing morphology. *Proc. R. Soc. B* **292**, 20251754 (2025).

25. A. Perrard, M. Baylac, J. M. Carpenter, C. Villemant, Evolution of wing shape in hornets: Why is the wing venation efficient for species identification? *J. Evol. Biol.* **27**, 2665–2675 (2014).

26. A. Perrard, F. Lopez-Osorio, J. M. Carpenter, Phylogeny, landmark analysis and the use of wing venation to study the evolution of social wasps (Hymenoptera: Vespidae: Vespinae). *Cladistics* **32**, 406–425 (2016).

27. M. Dehon, M. S. Engel, M. Gérard, A. M. Aytekin, G. Ghisbain, P. H. Williams, P. Rasmont, D. Michez, Morphometric analysis of fossil bumble bees (Hymenoptera, Apidae, Bombini) reveals their taxonomic affinities. *ZooKeys* **891**, 71–118 (2019).

28. I. Fadel, P. S. Milla Carmona, Y. Liu, E. J. Rayfield, P. C. J. Donoghue, Morphological constraints in hymenopteran forewings limit flight efficiency optimization. *R. Soc. Open Sci.* **12**, 250224 (2025).

29. B. N. Danforth, The evolution of hymenopteran wings: The importance of size. *J. Zool.* **218**, 247–276 (1989).

30. K. S. Perfilieva, Trends in evolution of ant wing venation (Hymenoptera, Formicidae). *Entomol. Rev.* **90**, 857–870 (2010).

31. V. Žikić, S. S. Stanković, A. Petrović, M. Ilić Milošević, Ž. Tomanović, C. P. Klingenberg, A. Ivanović, Evolutionary relationships of wing venation and wing size and shape in Aphidiinae (Hymenoptera: Braconidae). *Org. Divers. Evol.* **17**, 607–617 (2017).

32. T. Engels, D. Kolomenskiy, F. O. Lehmann, Flight efficiency is a key to diverse wing morphologies in small insects. *J. R. Soc. Interface* **18**, 20210518 (2021).

33. N. A. Lapina, S. E. Farisenkov, P. N. Petrov, A. A. Polilov, Effects of miniaturization on wing structure and flight mechanics in insects. *Entomol. Rev.* **101**, 1126–1141 (2021).

34. F. Ronquist, S. Klopfstein, L. Vilhelmsen, S. Schulmeister, D. L. Murray, A. P. Rasnitsyn, A total-evidence approach to dating with fossils, applied to the early radiation of the Hymenoptera. *Syst. Biol.* **61**, 973–999 (2012).

35. D. Adams, F. J. Rohlf, D. Slice, A field comes of age: Geometric morphometrics in the 21st century. *Hystrix Ital. J. Mammal.* **24**, 7–14 (2013).

36. M. Zelditch, D. Swiderski, H. D. Sheets, *Geometric Morphometrics for Biologists: A Primer* (Academic Press, 2012).

37. A. Goswami, A. Watanabe, R. N. Felice, C. Bardua, A. C. Fabre, P. D. Polly, High-density morphometric analysis of shape and integration: The good, the bad, and the not-really-a-problem. *Integr. Comp. Biol.* **59**, 669–683 (2019).

38. C. G. Northcutt, A. Athalye, J. Mueller, Pervasive label errors in test sets destabilize machine learning benchmarks. arXiv:2103.14749 [stat.ML] (2021).

39. C. González-Santoyo, D. Renza, E. Moya-Albor, Identifying and mitigating label noise in deep learning for image classification. *Technologies* **13**, 132 (2025).

40. M. Tsutsumi, N. Saito, D. Koyabu, C. Furusawa, A deep learning approach for morphological feature extraction based on variational auto-encoder: An application to mandible shape. *npj Syst. Biol. Appl.* **9**, 30 (2023).

41. A. Yakimovich, A. Beaugnon, Y. Huang, E. Ozkirimli, Labels in a haystack: Approaches beyond supervised learning in biomedical applications. *Patterns* **2**, 100383 (2021).

42. R. Dwivedi, D. Dave, H. Naik, S. Singhal, R. Omer, P. Patel, B. Qian, Z. Wen, T. Shah, G. Morgan, R. Ranjan, Explainable AI (XAI): Core ideas, techniques, and solutions. *ACM Comput. Surv.* **55**, 194 (2023).

43. T. G. Davies, I. A. Rahman, S. Lautenschlager, J. A. Cunningham, R. J. Asher, P. M. Barrett, K. T. Bates, S. Bengtson, R. B. J. Benson, D. M. Boyer, J. Braga, J. A. Bright, L. P. A. M. Claessens, P. G. Cox, X. P. Dong, A. R. Evans, P. L. Falkingham, M. Friedman, R. J. Garwood, A. Goswami, J. R. Hutchinson, N. S. Jeffery, Z. Johanson, R. Lebrun, C. Martínez-Pérez, J. Marugán-Lobón, P. M. O'Higgins, B. Metscher, M. Orliac, T. B. Rowe, M. Rücklin, M. R. Sánchez-Villagra, N. H. Shubin, S. Y. Smith, J. M. Starck, C. Stringer, A. P. Summers, M. D. Sutton, S. A. Walsh, V. Weisbecker, L. M. Witmer, S. Wroe, Z. Yin, E. J. Rayfield, P. C. J. Donoghue, Open data and digital morphology. *Proc. R. Soc. B Biol. Sci.* **284**, 20170194 (2017).

44. J. N. Keating, R. J. Garwood, R. S. Sansom, Phylogenetic congruence, conflict and consilience between molecular and morphological data. *BMC Ecol. Evol.* **23**, 30 (2023).

45. C. Jouault, N. Oyama, S. Álvarez-Parra, D. Huang, V. Perrichot, F. L. Condamine, F. Legendre, The radiation of Hymenoptera illuminated by Bayesian inferences from the fossil record. *Curr. Biol.* **35**, 2164–2174.e4 (2025).

46. J. F. Hoyal Cuthill, N. Guttenberg, S. Ledger, R. Crowther, B. Huertas, Deep learning on butterfly phenotypes tests evolution's oldest mathematical model. *Sci. Adv.* **5**, eaaw4967 (2019).

47. N. MacLeod, L. Kolska Horwitz, Machine-learning strategies for testing patterns of morphological variation in small samples: Sexual dimorphism in gray wolf (*Canis lupus*) crania. *BMC Biol.* **18**, 113 (2020).

48. T. Hauffe, J. L. Cantalapiedra, D. Silvestro, Trait-mediated speciation and human-driven extinctions in proboscideans revealed by unsupervised Bayesian neural networks. *Sci. Adv.* **10**, eadl2643 (2024).

49. C. R. Betts, R. J. Wootton, Wing shape and flight behavior in butterflies (Lepidoptera: Papilionoidea and Hesperioidea): A preliminary analysis. *J. Exp. Biol.* **138**, 271–288 (1988).

50. C. X. Zhang, J. A. Brisson, H. J. Xu, Molecular mechanisms of wing polymorphism in insects. *Annu. Rev. Entomol.* **64**, 297–314 (2019).

51. D. M. Linz, Y. Hu, A. P. Moczek, From descent with modification to the origins of novelty. *Zoology* **143**, 125836 (2020).

52. G. Forni, J. Martelossi, P. Valero, F. H. Hennemann, O. Conle, A. Luchetti, B. Mantovani, Macroevolutionary analyses provide new evidence of phasmid wings evolution as a reversible process. *Syst. Biol.* **71**, 1471–1486 (2022).

53. C. Le Roy, N. Tervelde, T. Engels, F. T. Muijres, Adaptations in wing morphology rather than wingbeat kinematics enable flight in small hoverfly species. *eLife* **13**, RP97839 (2025).

54. A. P. Rasnitsyn, Origin and evolution of Hymenoptera. *Tr. Paleontol. Inst. Akad. Nauk SSSR* **123**, 1–196 (1969).

55. A. P. Rasnitsyn, Origin and evolution of Hymenoptera. *Tr. Paleontol. Inst. Akad. Nauk SSSR* **174**, 1–192 (1980).

56. R. J. Wootton, Functional morphology of insect wings. *Annu. Rev. Entomol.* **37**, 113–140 (1992).

57. R. Dudley, *The Biomechanics of Insect Flight: Form, Function, Evolution* (Princeton Univ. Press, 2002).

58. B. B. Blaimer, B. F. Santos, A. Cruaud, M. W. Gates, R. R. Kula, I. Mikó, J.-Y. Rasplus, D. R. Smith, E. J. Talamas, S. G. Brady, M. L. Buffington, Key innovations and the diversification of Hymenoptera. *Nat. Commun.* **14**, 1212 (2023).

59. Y. M. Zhang, S. Bossert, T. Spasojevic, Evolving perspectives in Hymenoptera systematics: Bridging fossils and genomes across time. *Syst. Entomol.* **50**, 1–31 (2025).

60. M. Foote, The evolution of morphological diversity. *Annu. Rev. Ecol. Syst.* **28**, 129–152 (1997).

61. G. J. Slater, Not-so-early bursts and the dynamic nature of morphological diversification. *Proc. Natl. Acad. Sci. U.S.A.* **112**, 3595–3596 (2015).

62. L. E. M. Vet, Parasitoid searching efficiency links behavior to population processes. *Appl. Entomol. Zool.* **36**, 399–408 (2001).

63. L. Vilhelmsen, N. Isidoro, R. Romani, H. H. Basibuyuk, D. L. J. Quicke, Host location and oviposition in a basal group of parasitic wasps: The subgenual organ, ovipositor apparatus and associated structures in the Orussidae (Hymenoptera, Insecta). *Zoomorphology* **121**, 63–84 (2001).

64. J. P. Oeyen, P. Baa-Puyoulet, J. B. Benoit, L. W. Beukeboom, E. Bornberg-Bauer, A. Buttstedt, F. Calevro, E. I. Cash, H. Chao, H. Charles, M. J. M. Chen, C. Childers, A. G. Cridge, P. Dearden, H. Dinh, H. V. Doddapaneni, A. Dolan, A. Donath, D. Dowling, S. Dugan, E. Duncan, E. N. Elpidina, M. Friedrich, E. Geuverink, J. D. Gibson, S. Grath, C. J. P. Grimmelikhuijzen, E. Große-Wilde, C. Gudobba, Y. Han, B. S. Hansson, F. Hauser, D. S. T. Hughes, P. Ioannidis, E. Jacquin-Joly, E. C. Jennings, J. W. Jones, S. Klasberg, S. L. Lee, P. Lesný, M. Lovegrove, S. Martin, A. G. Martynov, C. Mayer, N. Montagné, V. C. Moris, M. Munoz-Torres, S. C. Murali, D. M. Muzny, B. Oppert, N. Parisot, T. Pauli, R. S. Peters, M. Petersen, C. Pick, E. Persyn, L. Podsiadlowski, M. F. Poelchau, P. Provataris, J. Qu, M. J. M. F. Reijnders, B. M. von Reumont, A. J. Rosendale, F. A. Simao, J. Skelly, A. G. Sotiropoulos, A. L. Stahl, M. Sumitani, E. M. Szuter, O. Tidswell, E. Tsitlakidis, L. Vedder, R. M. Waterhouse, J. H. Werren, J. Wilbrandt, K. C. Worley, D. S. Yamamoto, L. van de Zande, E. M. Zdobnov, T. Ziesmann, R. A. Gibbs, S. Richards, M. Hatakeyama, B. Misof, O. Niehuis,

Sawfly genomes reveal evolutionary acquisitions that fostered the mega-radiation of parasitoid and eusocial Hymenoptera. *Genome Biol. Evol.* **12**, 1099–1188 (2020).

65. R. S. Peters, L. Krogmann, C. Mayer, A. Donath, S. Gunkel, K. Meusemann, A. Kozlov, L. Podsiadlowski, M. Petersen, R. Lanfear, P. A. Diez, J. Heraty, K. M. Kjer, S. Klopfstein, R. Meier, C. Polidori, T. Schmitt, S. Liu, X. Zhou, T. Wappler, J. Rust, B. Misof, O. Niehuis, Evolutionary history of the Hymenoptera. *Curr. Biol.* **27**, 1013–1018 (2017).

66. C. Jouault, V. Ngô-Muller, J. M. Pouillon, A. Nel, New Burmese amber fossils clarify the evolution of bethylid wasps (Hymenoptera: Chrysidoidea). *Zool. J. Linn. Soc.* **191**, 1044–1058 (2021).

67. D. L. J. Quicke, *The Braconid and Ichneumonid Parasitoid Wasps: Biology, Systematics, Evolution and Ecology* (Wiley-Blackwell, 2014).

68. C. He, Y. Yang, X. Zhao, J. Li, Y. Cai, L. Peng, Y. Liu, S. Xiong, Y. Mei, Z. Yan, J. Wang, S. Xiao, Z. Teng, X. Gao, H. Xue, Q. Fang, G. Ye, X. Ye, Large-scale genome analyses provide insights into Hymenoptera evolution. *Mol. Biol. Evol.* **42**, msaf221 (2025).

69. M. G. Branstetter, B. N. Danforth, J. P. Pitts, B. C. Faircloth, P. S. Ward, M. L. Buffington, S. G. Brady, Phylogenomic insights into the evolution of stinging wasps and the origins of ants and bees. *Curr. Biol.* **27**, 1019–1025 (2017).

70. O. Sanisidro, M. C. Mihlbachler, J. L. Cantalapiedra, A macroevolutionary pathway to megaherbivory. *Science* **380**, 616–618 (2023).

71. Z. Guo, M. J. Benton, T. L. Stubbs, Z.-Q. Chen, Morphological innovation did not drive diversification in Mesozoic-Cenozoic brachiopods. *Nat. Ecol. Evol.* **8**, 1948–1958 (2024).

72. X. Liu, H. Song, D. Chu, X. Dai, F. Wang, D. Silvestro, Heterogeneous selectivity and morphological evolution of marine clades during the Permian-Triassic mass extinction. *Nat. Ecol. Evol.* **8**, 1248–1258 (2024).

73. E. O. Wilson, *The Insect Societies* (Harvard Univ. Press, 1971).

74. D. H. Erwin, Novelty and innovation in the history of life. *Curr. Biol.* **25**, R930–R940 (2015).

75. A. Matte, A. C. LeBoeuf, Innovation in ant larval feeding facilitated queen-worker divergence and social complexity. *Proc. Natl. Acad. Sci. U.S.A.* **122**, e2413742122 (2025).

76. B. N. Danforth, R. L. Minckley, J. L. Neff, *The Solitary Bees: Biology, Evolution, Conservation* (Princeton Univ. Press, 2019).

77. M. R. Richter, Social wasp (Hymenoptera: Vespidae) foraging behavior. *Annu. Rev. Entomol.* **45**, 121–150 (2000).

78. A. Fateryga, Trophic relations between vespid wasps (Hymenoptera, Vespidae) and flowering plants in the Crimea. *Entomol. Rev.* **90**, 698–705 (2010).

79. R. E. Brock, A. Cini, S. Sumner, Ecosystem services provided by aculeate wasps. *Biol. Rev.* **96**, 1645–1675 (2021).

80. B. Epstein, R. Meir, Generalization bounds for unsupervised and semi-supervised learning with autoencoders. arXiv:1902.01449 [stat.ML] (2019).

81. Y. Liang, X. Li, X. Huang, Z. Zhang, Y. Yao, "An automated data mining framework using autoencoders for feature extraction and dimensionality reduction" in *2024 4th International Conference on Electronic Information Engineering and Computer Communication* (EIECC, 2024), pp. 710–714.

82. M. Weiler, G. Cesa, General E(2)-equivariant steerable CNNs. *Adv. Neural Inf. Process. Syst.* **32**, 14357–14368 (2019).

83. J. Burgess, J. J. Deng, Orientation-invariant autoencoders learn robust representations for shape profiling of cells and organelles. *Nat. Commun.* **15**, 1022 (2024).

84. J. Ho, A. Jain, P. Abbeel, Denoising diffusion probabilistic models. *Adv. Neural Inf. Process. Syst.* **33**, 6840–6851 (2020).

85. T. Karras, S. Laine, M. Aittala, J. Hellsten, J. Lehtinen, T. Aila, “Analyzing and improving the image quality of StyleGAN” in *Proceeding IEEE/CVF Conference Computer Vision Pattern Recognition* (IEEE, 2020), pp. 8107–8116.

86. R. Rombach, A. Blattmann, D. Lorenz, P. Esser, B. Ommer, “High-resolution image synthesis with latent diffusion models” in *Proceeding IEEE/CVF Conference Computer Vision Pattern Recognition* (IEEE, 2022), pp. 10684–10695.

87. A. Kirillov, E. Mintun, N. Ravi, H. Mao, C. Rolland, L. Gustafson, T. Xiao, S. Whitehead, A. C. Berg, W.-Y. Lo, P. Dollár, R. Girshick, “Segment anything” in *Proceeding IEEE/CVF International Conference on Computer Vision* (IEEE, 2023), pp. 3992–4003.

88. G. E. Hinton, R. R. Salakhutdinov, Reducing the dimensionality of data with neural networks. *Science* **313**, 504–507 (2006).

89. D. P. Kingma, M. Welling, “Auto-encoding variational Bayes” in *Conference proceedings: papers accepted to the International Conference on Learning Representations* (ICLR, 2014).

90. H. Kobayashi, K. C. Cheveralls, M. D. Leonetti, L. A. Royer, Self-supervised deep learning encodes high-resolution features of protein subcellular localization. *Nat. Methods* **19**, 995–1003 (2022).

91. S. Ioffe, C. Szegedy, “Batch normalization: Accelerating deep network training by reducing internal covariate shift” in *Proceeding 32nd International Conference Machine Learning* (2015), pp. 448–456.

92. V. Nair, G. E. Hinton, “Rectified linear units improve restricted Boltzmann machines” in *Proceeding 27th International Conference Machine Learning* (2010), pp. 807–814.

93. M. Lin, Q. Chen, S. Yan, “Network in network” in *Conference proceedings: papers accepted to the International Conference on Learning Representations* (ICLR, 2014).

94. I. Loshchilov, F. Hutter, “Decoupled weight decay regularization” in *7th International Conference on Learning Representations* (ICLR, 2019).

95. C. M. Bishop, *Pattern Recognition and Machine Learning* (Springer, 2006).

96. A. E. Hoerl, R. W. Kennard, Ridge regression: Biased estimation for nonorthogonal problems. *Technometrics* **12**, 55–67 (1970).

97. Z. Wu, S. Song, A. Khosla, F. Yu, L. Zhang, X. Tang, J. Xiao, “3D ShapeNets: A deep representation for volumetric shapes” in *Proceeding IEEE/CVF Conference Computer Vision Pattern Recognition* (IEEE, 2015), pp. 1912–1920.

98. S. Almécija, K. D. Pugh, A. Anaya, C. M. Smith, N. B. Simmons, R. S. Voss, S. A. Catalano, Primate phenotypes: A multi-institution collection of 3D morphological data housed in MorphoSource. *Sci. Data* **11**, 1391 (2024).

99. J. MacQueen, Some methods for classification and analysis of multivariate observations. *Berkeley Symp. Math. Stat. Probab.* **1**, 281–297 (1967).

100. K. Pearson, On lines and planes of closest fit to systems of points in space. *Philos. Mag.* **2**, 559–572 (1901).

101. L. Van der Maaten, G. Hinton, Visualizing data using *t*-SNE. *J. Mach. Learn. Res.* **9**, 2579–2605 (2008).

102. P. J. Rousseeuw, Silhouettes: A graphical aid to the interpretation and validation of cluster analysis. *J. Comput. Appl. Math.* **20**, 53–65 (1987).

103. T. Caliński, J. Harabasz, A dendrite method for cluster analysis. *Commun. Stat.* **3**, 1–27 (1974).

104. D. L. Davies, D. W. Bouldin, A cluster separation measure. *IEEE Trans. Pattern Anal. Mach. Intell.* **1**, 224–227 (1979).

105. K. Simonyan, A. Zisserman, “Very deep convolutional networks for large-scale image recognition” in *3rd International Conference on Learning Representations* (ICLR, 2015).

106. K. He, X. Zhang, S. Ren, J. Sun, "Deep residual learning for image recognition" in *Proceeding IEEE/CVF Conference Computer Vision Pattern Recognition* (IEEE, 2016), pp. 770–778.

107. G. Huang, Z. Liu, L. Van Der Maaten, K. Q. Weinberger, "Densely connected convolutional networks" in *Proceeding IEEE/CVF Conference Computer Vision Pattern Recognition* (IEEE, 2017), pp. 4700–4708.

108. F. Chollet, "Xception: Deep learning with depthwise separable convolutions" in *Proceeding IEEE/CVF Conference Computer Vision Pattern Recognition* (IEEE, 2017), pp. 1251–1258.

109. C. Szegedy, V. Vanhoucke, S. Ioffe, J. Shlens, Z. Wojna, "Rethinking the inception architecture for computer vision" in *Proceeding IEEE/CVF Conference Computer Vision Pattern Recognition* (IEEE, 2016), pp. 2818–2826.

110. C. Szegedy, S. Ioffe, V. Vanhoucke, A. A. Alemi, Inception-v4, inception-resnet and the impact of residual connections on learning. *Proc. AAAI Conf. Artif. Intell.* **31**, 4278–4284 (2017).

111. M. Tan, Q. V. Le, "Efficientnet: Rethinking model scaling for convolutional neural networks" in *International Conference Machine Learning* (PMLR, 2019), pp. 6105–6114.

112. M. Sandler, A. Howard, M. Zhu, A. Zhmoginov, L. C. Chen, "Mobilenetv2: Inverted residuals and linear bottlenecks" in *Proceeding IEEE/CVF Conference Computer Vision Pattern Recognition* (IEEE, 2018), pp. 4510–4520.

113. B. Zoph, V. Vasudevan, J. Shlens, Q. V. Le, "Learning transferable architectures for scalable image recognition" in *Proceeding IEEE/CVF Conference Computer Vision Pattern Recognition* (IEEE, 2018), pp. 8697–8706.

114. J.-B. Grill, F. Strub, F. Altché, C. Tallec, P. H. Richemond, E. Buchatskaya, C. Doersch, B. A. Pires, Z. D. Guo, M. G. Azar, B. Piot, K. Kavukcuoglu, R. Munos, M. Valko, Bootstrap your own latent: A new approach to self-supervised learning. *Adv. Neural Inf. Process. Syst.* **33**, 21271–21284 (2020).

115. T. Chen, S. Kornblith, M. Norouzi, G. Hinton, “A simple framework for contrastive learning of visual representations” in *International Conference Machine Learning* (2020), pp. 1597–1607.

116. K. He, H. Fan, Y. Wu, S. Xie, R. Girshick, “Momentum contrast for unsupervised visual representation learning” in *Proceeding IEEE/CVF Conference Computer Vision Pattern Recognition* (IEEE, 2020), pp. 9729–9738.

117. A. Chattopadhay, A. Sarkar, P. Howlader, V. N. Balasubramanian, “Grad-CAM++: Generalized gradient-based visual explanations for deep convolutional networks” in *Proceeding 2018 IEEE Winter Conference Application Computer Vision* (WACV, 2018), pp. 839–847.

118. S. Abnar, W. Zuidema, “Quantifying attention flow in transformers” in *Proceeding 58th Annual Meeting Association Computational Linguistic* (ACL 2020), pp. 4190–4197.

119. A. Nel, J. Prokop, P. Nel, P. Grandcolas, D.-Y. Huang, P. Roques, E. Guilbert, O. Dostál, J. Szwedo, Traits and evolution of wing venation pattern in paraneopteran insects. *J. Morphol.* **273**, 480–506 (2012).

120. B. Misof, S. Liu, K. Meusemann, R. S. Peters, A. Donath, C. Mayer, P. B. Frandsen, J. Ware, T. Flouri, R. G. Beutel, O. Niehuis, M. Petersen, F. Izquierdo-Carrasco, T. Wappler, J. Rust, A. J. Aberer, U. Aspöck, H. Aspöck, D. Bartel, A. Blanke, S. Berger, A. Böhm, T. R. Buckley, B. Calcott, J. Chen, F. Friedrich, M. Fukui, M. Fujita, C. Greve, P. Grobe, S. Gu, Y. Huang, L. S. Jermiin, A. Y. Kawahara, L. Krogmann, M. Kubiak, R. Lanfear, H. Letsch, Y. Li, Z. Li, J. Li, H. Lu, R. Machida, Y. Mashimo, P. Kapli, D. D. McKenna, G. Meng, Y. Nakagaki, J. L. Navarrete-Heredia, M. Ott, Y. Ou, G. Pass, L. Podsiadlowski, H. Pohl, B. M. von Reumont, K. Schütte, K. Sekiya, S. Shimizu, A. Slipinski, A. Stamatakis, W. Song, X. Su, N. U. Szucsich, M. Tan, X. Tan, M. Tang, J. Tang, G. Timelthaler, S. Tomizuka, M. Trautwein, X. Tong, T. Uchifune, M. G. Walzl, B. M. Wiegmann, J. Wilbrandt, B. Wipfler, T. K. F. Wong, Q. Wu, G. Wu, Y. Xie, S. Yang, Q. Yang, D. K. Yeates, K. Yoshizawa, Q. Zhang, R. Zhang, W. Zhang, Y. Zhang, J. Zhao, C. Zhou, L. Zhou, T. Ziesmann, S. Zou, Y. Li, X. Xu, Y. Zhang, H. Yang, J. Wang, J. Wang, K. M. Kjer, X. Zhou, Phylogenomics resolves the timing and pattern of insect evolution. *Science* **346**, 763–767 (2014).

121. B. Wipfler, H. Letsch, P. B. Frandsen, P. Kapli, C. Mayer, D. Bartel, T. R. Buckley, A. Donath, J. S. Edgerly-Rooks, M. Fujita, S. Liu, R. Machida, Y. Mashimo, B. Misof, O. Niehuis, R. S. Peters, M. Petersen, L. Podsiadlowski, K. Schütte, S. Shimizu, T. Uchifune, J. Wilbrandt, E. Yan, X. Zhou, S. Simon, Evolutionary history of Polyneoptera and its implications for our understanding of early winged insects. *Proc. Natl. Acad. Sci. U.S.A.* **116**, 3024–3029 (2019).

122. M. J. Anderson, Distance-based tests for homogeneity of multivariate dispersions. *Biometrics* **62**, 245–253 (2006).

123.. Hammer, D. A. T. Harper, P. D. Ryan, PAST: Paleontological Statistics Software Package for Education and Data Analysis. *Palaeontol. Electron.* **4**, 1–9 (2001).

124. P. A. Goloboff, M. E. Morales, TNT version 1.6, with a graphical interface for MacOS and Linux, including new routines in parallel. *Cladistics* **39**, 144–153 (2023).

125. A. A. Polilov, *At the Size Limit-Effects of Miniaturization in Insects* (Springer, 2016).

126. R. Guidotti, A. Monreale, S. Ruggieri, F. Turini, F. Giannotti, D. Pedreschi, A survey of methods for explaining black box models. *ACM Comput Surv* **51**, 1–42 (2019).

127. H. Goulet, J. T. Huber, Hymenoptera of the World: An Identification Guide to Families (Research Branch Agriculture Canada Publication 1894/E, 1993).

128. A. V. Antropov, “Body Size Diminution and the Ways of Evolution of Wing Venation in Aculeate Hymenopterous Insects (Insecta: Hymenoptera: Aculeata: Apoidea). An Attempt of Functional Interpretation” in *Evolution and Systematics: Lamarck and Darwin in Modern Studies*, A. V. Sviridov, A. I. Shatalkin, Eds. (Archiv of Zoology of the Museum of Moscow University, 2009), vol. 50, pp. 247–274.

129. A. Perrard, Wasp waist and flight: Convergent evolution in wasps reveals a link between wings and body shapes. *Am. Nat.* **195**, 181–191 (2020).

130. S. P. Blomberg, T. Garland Jr., A. R. Ives, Testing for phylogenetic signal in comparative data: Behavioral traits are more labile. *Evolution* **57**, 717–745 (2003).

131. M. Pagel, The maximum likelihood approach to reconstructing ancestral character states of discrete characters on phylogenies. *Syst. Biol.* **48**, 612–622 (1999).

132. L. J. Revell, phytools: An R package for phylogenetic comparative biology (and other things). *Methods Ecol. Evol.* **3**, 217–223 (2012).

133. R. Ihaka, R. Gentleman, R: A language for data analysis and graphics. *J. Comput. Graph. Stat.* **5**, 299–314 (1996).

134. E. Paradis, K. Schliep, ape 5.0: An environment for modern phylogenetics and evolutionary analyses in R. *Bioinformatics* **35**, 526–528 (2019).

135. M. W. Pennell, J. M. Eastman, G. J. Slater, J. W. Brown, J. C. Uyeda, R. G. FitzJohn, M. E. Alfaro, L. J., Harmon, geiger v2.0: An expanded suite of methods for fitting macroevolutionary models to phylogenetic trees. *Bioinformatics* **30**, 2216–2218 (2014).

136. H. Wickham, *ggplot2: Elegant Graphics for Data Analysis* (Springer-Verlag, 2016).

137. A. Dosovitskiy, L. Beyer, A. Kolesnikov, D. Weissenborn, X. Zhai, T. Unterthiner, M. Dehghani, M. Minderer, G. Heigold, S. Gelly, J. Uszkoreit, N. Houlsby, "An image is worth 16×16 words: Transformers for image recognition at scale" in *9th International Conference on Learning Representations* (ICLR, 2021).
